# GPCRs Direct Germline Development and Somatic Gonad Function in Planarians

**DOI:** 10.1371/journal.pbio.1002457

**Published:** 2016-05-10

**Authors:** Amir Saberi, Ayana Jamal, Isabel Beets, Liliane Schoofs, Phillip A. Newmark

**Affiliations:** 1 Howard Hughes Medical Institute and Department of Cell and Developmental Biology, University of Illinois at Urbana-Champaign, Urbana, Illinois, United States of America; 2 Department of Biology, Functional Genomics and Proteomics Unit, KU Leuven, Leuven, Belgium; Cornell University, UNITED STATES

## Abstract

Planarians display remarkable plasticity in maintenance of their germline, with the ability to develop or dismantle reproductive tissues in response to systemic and environmental cues. Here, we investigated the role of G protein-coupled receptors (GPCRs) in this dynamic germline regulation. By genome-enabled receptor mining, we identified 566 putative planarian GPCRs and classified them into conserved and phylum-specific subfamilies. We performed a functional screen to identify NPYR-1 as the cognate receptor for NPY-8, a neuropeptide required for sexual maturation and germ cell differentiation. Similar to NPY-8, knockdown of this receptor results in loss of differentiated germ cells and sexual maturity. NPYR-1 is expressed in neuroendocrine cells of the central nervous system and can be activated specifically by NPY-8 in cell-based assays. Additionally, we screened the complement of GPCRs with expression enriched in sexually reproducing planarians, and identified an orphan chemoreceptor family member, *ophis*, that controls differentiation of germline stem cells (GSCs). *ophis* is expressed in somatic cells of male and female gonads, as well as in accessory reproductive tissues. We have previously shown that somatic gonadal cells are required for male GSC specification and maintenance in planarians. However, *ophis* is not essential for GSC specification or maintenance and, therefore, defines a secondary role for planarian gonadal niche cells in promoting GSC differentiation. Our studies uncover the complement of planarian GPCRs and reveal previously unappreciated roles for these receptors in systemic and local (i.e., niche) regulation of germ cell development.

## Abstract 2

Genome-wide analysis of the planarian Schmidtea mediterranea reveals a complement of over 550 G protein-coupled receptors, including two with critical roles in germline development.

## Author Summary

G protein-coupled receptors (GPCRs) are the largest and most versatile family of cell-surface receptors. They play critical roles in various cellular and physiological systems and have emerged as a leading group of therapeutic targets. Due to their structural and functional conservation across animals, much has been learned about GPCRs from studies in laboratory models. Here, we performed genome-wide receptor mining to identify and categorize the complement of GPCR-encoding genes in the planarian *Schmidtea mediterranea*, an emerging model organism for regeneration and germ cell biology. We then conducted two studies implicating planarian GPCRs in the regulation of reproductive function. First, we found the receptor component of a central neuropeptide Y signaling pathway and demonstrated its involvement in the systemic control of reproductive development. Next, we showed that a novel chemoreceptor family member is expressed in somatic cells of the planarian gonads and directs germ cell maturation via the niche. We predict that future studies on the hundreds of other planarian GPCRs identified in this work will not only help us understand the conserved role of these receptors in various physiological pathways but also pave the way for identification of novel therapeutic targets in parasitic relatives of the planarian.

## Introduction

G protein-coupled receptors (GPCRs) play critical roles in sexual reproduction, guiding germ cell migration, mediating hormonal regulation of gamete development, and facilitating the function of accessory reproductive tissues. For example, a complex network of peptidergic neurons in the mammalian hypothalamus controls the release of pituitary gonadotropins that systemically regulate gonadal function. GPCRs mediate various short- and long-range communication events in this hormonal cascade, whether the target is another neuron or a gonadal cell. Mutations in several of these GPCRs and their ligands are associated with hypogonadotropic hypogonadism and other reproductive disorders [1]. GPCRs that act as receptors for follicle-stimulating hormone (FSH), luteinizing hormone (LH), gonadotropin-releasing hormone (GnRH), kisspeptin, prokineticin, and tachykinin play essential roles in systemic regulation of gonadal function in mammals [2–6]. Despite extensive genetic information and molecular studies in mammalian models, much remains to be learned about the role of GPCR signaling in sensing physiological and environmental cues and the evolutionary conservation of these mechanisms in regulating reproduction across metazoans.

GPCRs also facilitate germ cell differentiation and maturation cell autonomously. Chemokine receptor CXCR4 in vertebrates and rhodopsin-like GPCR Tre1 in *Drosophila* enable chemokine-guided migration of primordial germ cells (PGCs) early in development [7–9]. Other olfactory, adhesion-like, and secretin-like GPCRs and their signaling partners are required for the maintenance and proliferation of germline progenitors and stem cells as well as gamete morphogenesis [10–15]. In many cases, however, GPCR ligands, signaling partners, and functional mechanisms are far from understood.

Platyhelminthes (flatworms) exhibit the remarkable ability to coordinate their reproductive development with systemic and environmental cues such as body size, nutritional status, and season. Upon starvation or severe injury, planarians are capable of reversibly disassembling their reproductive system, presumably to curb metabolic demand or to prepare for body-wide tissue remodeling [16]. Upon amputations that entirely remove the reproductive system, the remaining head fragments can re-specify germ cells and reproductive structures de novo [17,18]. Furthermore, a number of classic and recent studies suggest that planarian neuroendocrine cells systemically influence reproductive development. For example, head amputation, which involves removal of the cephalic ganglia, results in regression of the male gonads to clusters of PGCs [19,20]. Owing to its reproductive plasticity and the availability of numerous functional genomic tools, we use the planarian *Schmidtea mediterranea* as a model to study regulation of germ cell development and reproductive function, focusing here on the GPCR superfamily.

Flatworm GPCRs have been the focus of very few functional studies, which have been limited mainly to neurotransmitter response, body patterning through the Wnt/frizzled pathway, or photoreception by opsins [21–25]. An earlier genome-wide study of flatworm GPCRs was based on an incomplete genome assembly and limited to in silico prediction of GPCR genes [26]. While planarian neuropeptides and other GPCR ligands have received some attention [27–31], receptor/ligand pairs and their specific physiological function in planarians have not been defined. Our recent genome-wide characterization of planarian neuropeptides identified NPY-8, a conserved neuropeptide Y homolog, that is required for proper development of the planarian germline [32]. Here, we characterized the planarian GPCR complement to identify the NPY-8 receptor and other GPCRs involved in regulating germ cell development. Our studies suggest that GPCRs within the central nervous system (CNS) and the gonads are key components of the signal transduction mechanisms that regulate reproductive development in planarians.

## Results

### Genome-Wide Analysis Reveals Conserved and Phylum-Specific GPCR Families

To explore the role of GPCRs in planarian reproductive biology, we generated a comprehensive database of planarian GPCR gene sequences, classes, and expression information. A previous annotation of flatworm GPCRs was based on an early draft of the planarian genome [26]. We found that many GPCR genes are absent from this list, while several genes encode proteins that more closely resemble other transmembrane protein families. To generate a complete database, we used extensive transcriptomic data to assemble a de novo transcriptome that we mined for putative seven-transmembrane receptor sequences (workflow shown in S1A Fig, de novo transcriptome can be found in S1 Data). Combining these transcriptomic data and sensitive pattern-discovery methods, we developed a comprehensive list of 566 GPCRs. We confirmed and improved the annotation of 343 previously identified GPCRs and discovered 223 new ones (S2 and S3 Data). The availability of RNA-seq data from specific tissues or experimentally modified planarians revealed a great deal of information about GPCR expression in planarians (S3 Data, S1D Fig). A partial in situ hybridization (ISH) screen revealed expression of planarian GPCRs in a variety of tissues, including nervous and reproductive systems, intestine, epithelium, and presumptive sensory organs (S2 Fig).

**Fig 1:**
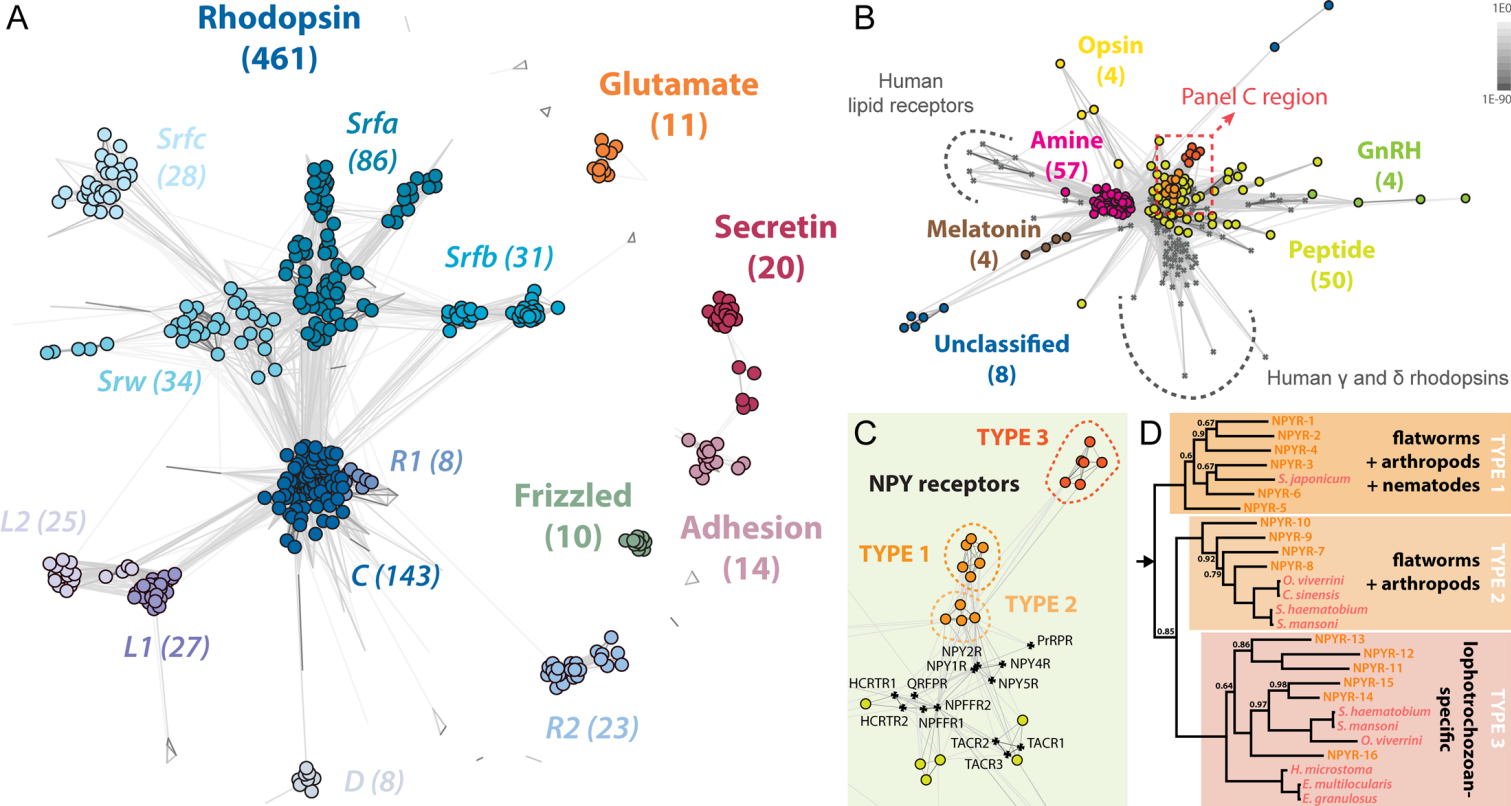
Global view of planarian GPCRs and the NPY receptor family. (A) Similarity clustering of 566 planarian GPCRs revealing members of all five metazoan GPCR families. Only GPCRs of the convex clusters are counted and highlighted by filled circles (see Materials and Methods). (B) Co-clustering of conserved planarian and human rhodopsin-like GPCRs reveals large groups of amine and peptide receptors, as well as smaller groups of receptors with other functionalities. Planarians appear to lack homologs of human γ and δ rhodopsins or lipid receptors. Planarian *Rho-C* and human receptors are shown by filled circles and grey crossmarks, respectively. Darker edges indicate higher similarity (lower *p*-value) between nodes. *p*-value scale shown at top-right corner. Area in dashed box is expanded in panel C. (C) Magnified view of the similarity network around NPY receptors (dashed box in B). Three groups of planarian receptors (16 total) are located adjacent to human NPY receptors. (D) Bayesian inference topology of candidate planarian NPY receptors (orange) and parasitic flatworm homologs (pink). Arrow indicates the root. Posterior probabilities are 1.00 at every node, except those with a value shown. Three monophyletic groups of NPY receptors are found that are parallel to groups identified by similarity clustering in panel C. Type 1 is conserved across flatworms, arthropods, and nematodes, type 2 receptors are found in flatworms and arthropods, and type 3 receptors are lophotrochozoan-specific. The complete phylogenetic analysis is shown in S1E Fig. See S1 and S2 Figs for more information on the planarian GPCR complement.

To classify the complement of planarian GPCRs, we performed clustering analyses based on pairwise sequence similarities. This method constructs a graph in which nodes represent individual proteins and edges provide attractive forces proportional to the sequence similarity between protein pairs. Once the graph is optimized, groups of similar proteins aggregate into convex clusters that can be traced computationally [33]. This method has been used successfully to infer evolutionary relatedness between highly diverse GPCRs [34]. Sequence clustering analysis indicated that the planarian genome encodes receptors belonging to the five conserved metazoan GPCR classes: glutamate, rhodopsin, adhesion, frizzled, and secretin (GRAFS; Figs 1A and S1B) [35]. The 461-member planarian rhodopsin-like family (Figs 1A and S1B, left) is expanded beyond the conserved rhodopsin-like receptors, but its members still maintain the characteristic (D/E)R(Y/F) motif of rhodopsin-like GPCRs (S1C Fig). Only 143 of these rhodopsin-like GPCRs are conserved across all metazoans (*Rho-C*) and are located in the center of the rhodopsin family graph (Fig 1A, left). Co-clustering of *Rho-C* family members and human rhodopsin-like GPCRs revealed that planarians have homologs of human α- and β-group GPCRs (e.g., amine and peptide receptors, opsins) but not γ-group (including chemokine receptors), δ-group (including olfactory and purine receptors), or lipid receptors (Fig 1B) [35].

Other rhodopsin-like families include *Rho-L*, *Rho-R*, and *Rho-D* (arbitrary designations) that have no known homologs outside of platyhelminthes. The largest and most expanded rhodopsin-like family, *Srf/w* (for “Serpentine receptors of flatworms and srw”), contains 199 genes in four subclusters: *Srfa*, *Srfb*, and *Srfc* that appear to be flatworm-specific, and *Srw*, members of which aggregate with the *srw* subfamily of *Caenorhabditis elegans* chemoreceptors (S1B Fig). In *C*. *elegans*, members of the *srw* subfamily are the only chemoreceptors with a recognizable sequence similarity to the main rhodopsin family (specifically to FMRFamide receptors) [36]. While only one *C*. *elegans* chemoreceptor has been experimentally de-orphanized [37], GPCRs of this family are called “chemoreceptors” because a large fraction of them are required for chemosensation and/or expressed in chemosensory neurons that respond to environmental molecules [38]. For simplicity, we call the *Srf/w* family, which includes *ophis* (GB: KX018822, see below), the planarian chemoreceptor family.

A subset of putative planarian GPCRs (48 rhodopsin-like and 50 others) that did not join any convex clusters include homologs of conserved adiponectin receptors, lung seven-transmembrane receptors, leucine-rich repeat-containing GPCRs, and receptors with no known homologs (S3 Data, not highlighted by a circle in Fig 1A). Overall, our analyses reveal that major groups of planarian GPCRs fit within the conserved GRAFS classification; however, the rhodopsin-like family in planarians is highly diversified and includes multiple invertebrate-specific and potentially flatworm-specific subfamilies.

### NPY Receptor *npyr-1* Is Required for Systemic Regulation of Germline Development

**Fig 2:**
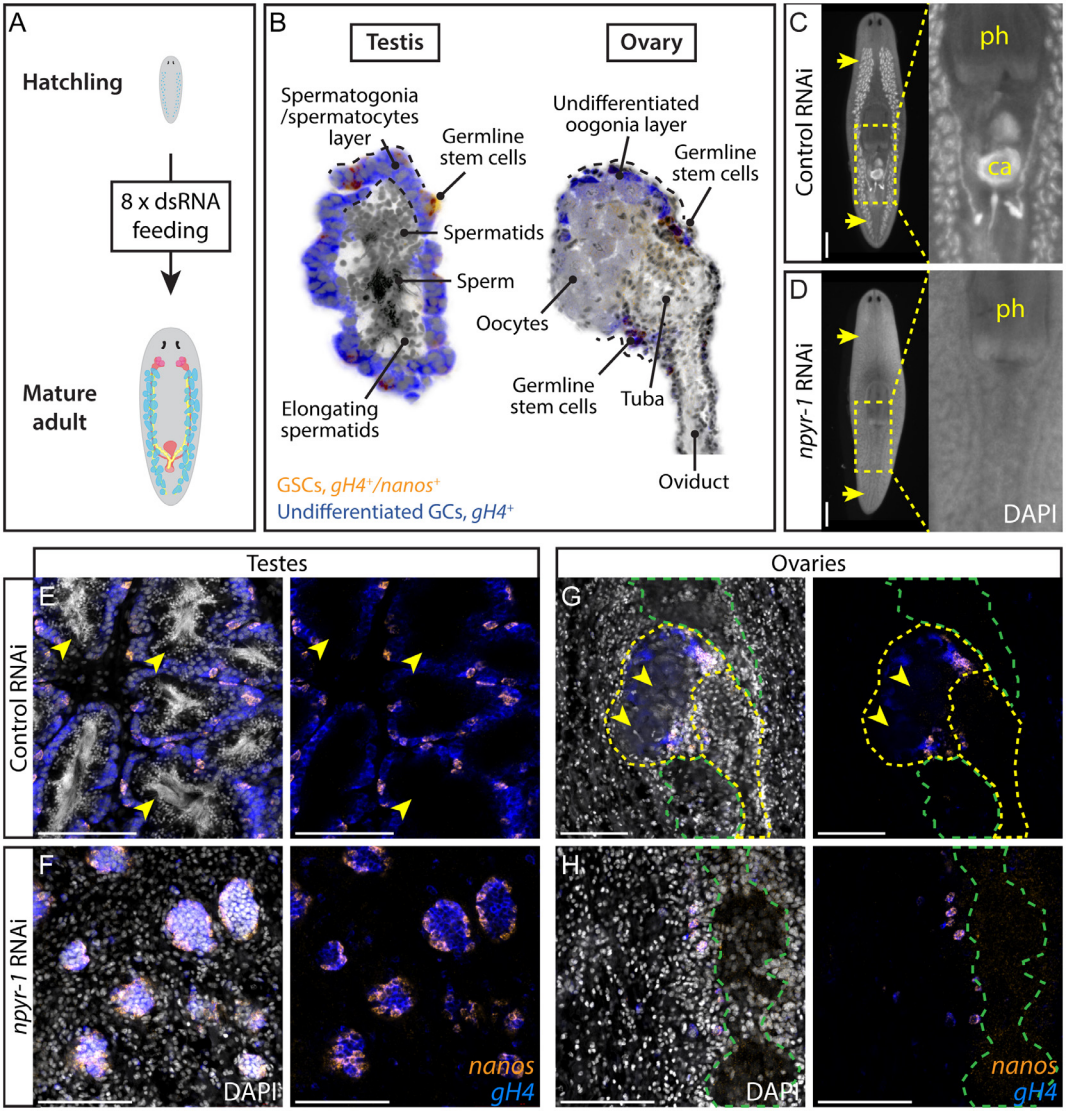
NPY receptor *npyr-1* is required for germ cell maturation. (A) Post-embryonic development RNAi paradigm used to determine the function of genes during normal planarian growth. Hatchlings (≤2 wk old) were fed dsRNA corresponding to each gene eight times to ensure that control worms achieve sexual maturity. (B) Schematic showing planarian testis and ovary structures. In both testes and ovaries, *nanos*^*+*^*/gH4*^*+*^ GSCs (orange) and *nanos*^*-*^*/gH4*^*+*^ spermatogonia/oogonia (blue) are located on the periphery, while more differentiated spermatids, sperm, or oocytes (grey) are in the middle of the gonads. (C, D) DAPI staining showing testes and stored sperm in whole-mount samples. Insets show the copulatory apparatus region. Pharynx and copulatory apparatus are marked by “ph” and “ca”, respectively. RNAi treatment followed the paradigm in A. Control worms (C) develop a complete reproductive system, while *npyr-1(RNAi)* worms (D) lack developed reproductive tissues and mature gametes. *n* = 5/5 for each of the three *npyr-1* clones and control. RNAi quantification data can be found in S4 Data. (E–H) Double-FISH labeling GSCs (*nanos*^*+*^*/gH4*^*+*^, orange) and spermatogonia (*nanos*^*-*^*/gH4*^*+*^, blue) in whole-mount control and *npyr-1(RNAi)* samples. GSCs and spermatogonial cells are present in both conditions. Testis germ cells differentiate into sperm and spermatids (arrowheads) in control worms (E), but not in *npyr-1(RNAi)* worms (F). In ovaries, mature oocytes with large cytoplasm (arrowheads) are surrounded by *gH4*^*+*^ oogonia in control planarians (G), but are absent in *npyr-1(RNAi)* animals (H). DAPI (grey) labels nuclei. Yellow dashed lines indicate ovaries and oviducts in G and H. Green dashed lines indicate cephalic ganglia near the ovaries. Scale bars are 1 mm in C and D and 100 μm in E–H. See also S3 Fig.

We next wanted to know if planarian GPCRs are involved in signaling pathways that regulate development and maintenance of the germline. Our previous studies have shown that *Smed-npy-8*, an NPY homolog expressed in the planarian nervous system, is required for germ cell development and sexual maturity. RNA interference (RNAi) knockdown of *npy-8* during post-embryonic development (paradigm in Fig 2A) blocks differentiation of both male and female germ cells, as well as formation of somatic accessory reproductive structures (S3A Fig) [32]. Our identification of the planarian GPCR repertoire allowed us to investigate the role of NPY receptors in planarian reproductive development. We hypothesized that if NPY-8 acts through a conserved NPY receptor to promote reproductive development, RNAi knockdown of at least one NPY receptor should phenocopy *npy-8(RNAi)*, barring functional redundancy.

Since NPY receptors are conserved throughout metazoans, to identify planarian NPY receptor genes we focused on *Rho-C* and repeated the clustering analysis only using planarian and human rhodopsin-like GPCRs (Fig 1B). Our analysis identified 16 putative planarian NPY receptors that cluster with human NPY receptors (Fig 1C). Bayesian phylogenetic analyses suggest that planarian NPY receptors exist in three monophyletic groups: one that includes flatworm, arthropod (including *Drosophila* NPFR-1 [39]), and nematode sequences, one that only includes flatworm and arthropod NPY receptors, and a third group that appears to be lophotrochozoan-specific (including the snail NPY receptor GRL105 [40]) (Figs 1D and S1E). Colorimetric ISH shows that most planarian NPY receptors are expressed in the CNS and the testes (S2H–S2P Fig).

To identify a candidate receptor for NPY-8, we individually knocked down each of the NPY receptor genes in planarian hatchlings (S3 Data, Fig 2A). *Smed-germinal histone H4* (*gH4*) was used as a marker for spermatogonial cells, female germ cells, and neoblasts, and *Smed-nanos* (*nanos*) was used to label male and female germline stem cells (GSCs) [18,41,42]. Knockdown of *Smed-npyr-1* (GB: KX018969) prevented germ cell differentiation and formation of the reproductive system, regardless of the region of the gene targeted (Fig 2C and 2D, S4 Data). In control treatments, testes reach maturity and produce sperm with compact, elongated nuclei readily visualized by DAPI staining (Fig 2E). In *npyr-1(RNAi)* planarians, testes only contain undifferentiated GSCs (*nanos*^*+*^*/gH4*^*+*^) and spermatogonia (*nanos*^*–*^*/gH4*^*+*^) (Fig 2F). *npyr-1* is also essential for female germ cell differentiation, as the ovaries in *npyr-1(RNAi)* worms only include GSCs and oogonia and lack mature oocytes seen in control worms (Fig 2G and 2H). Notably, the *nanos*^*+*^ GSC pool was present in all RNAi animals (Fig 2E–2H) and *nanos* mRNA expression was unaffected as assayed by quantitative PCR (qPCR)(S3C Fig). We also found that *npyr-1* is not required for de novo germ cell specification (S3B Fig).

Next, we wanted to rule out the possibility that the phenotypes observed after *npy-8* or *npyr-1* RNAi are an indirect consequence of down-regulating the other gene. We knocked down *npy-8* or *npyr-1* and found that while the targeted genes are down-regulated at least 4-fold, expression of the other gene is not affected (S3C Fig). Collectively, our experiments indicate that *npyr-1* knockdown phenotypes closely resemble those of *npy-8*, suggesting that *npyr-1* serves as an NPY-8 receptor to systemically regulate development of the germ cells into mature gametes.

### NPY-8 Specifically Activates NPYR-1 In Vitro

**Fig 3:**
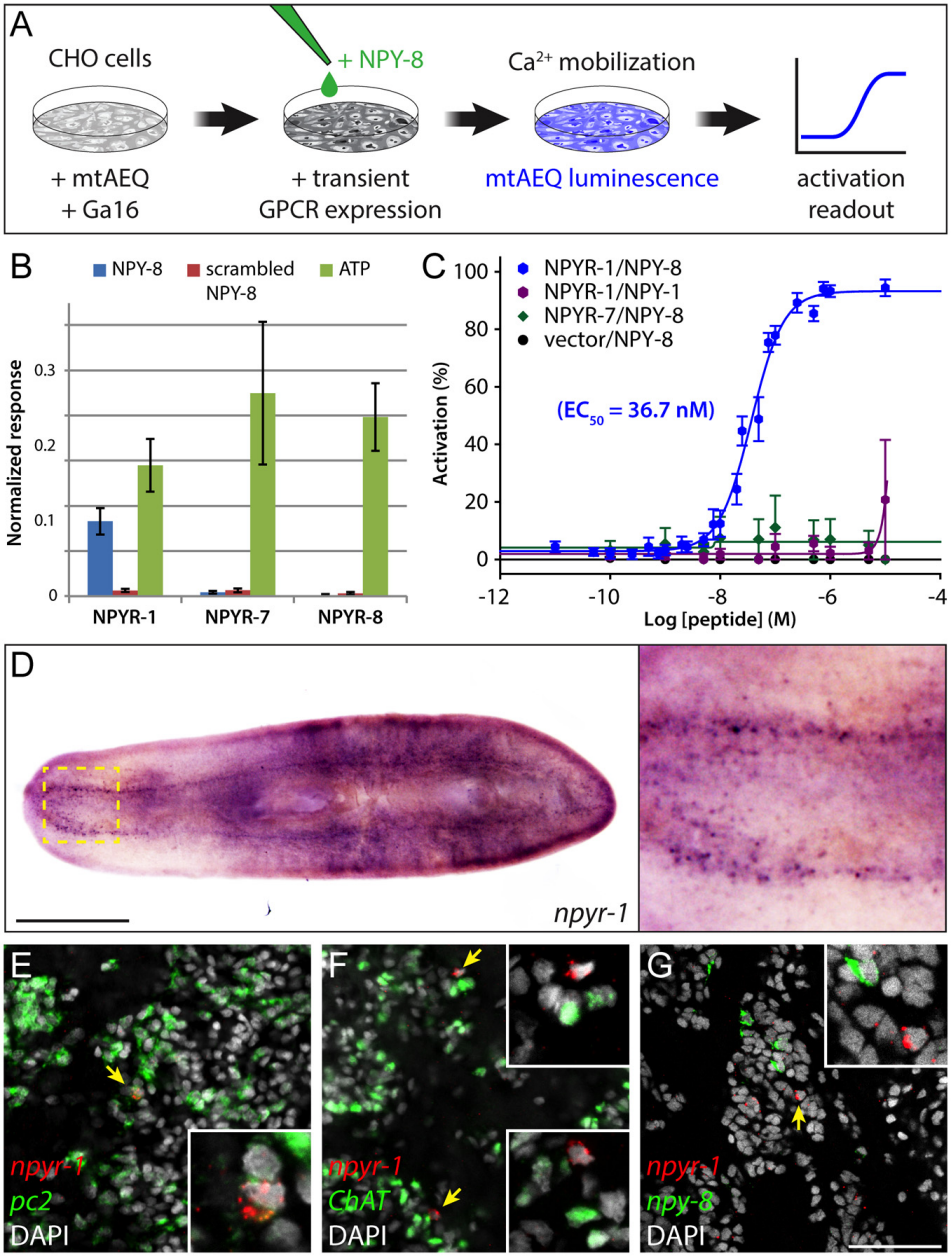
NPY-8 targets *npyr-1*^*+*^ neuroendocrine cells in the CNS. (A) Schematic of receptor-activation assay performed in CHO/mtAEQ/G16 cells. Candidate GPCRs were expressed transiently in a cell line that enables visualization of calcium mobilization upon receptor activation. (B) Normalized activation response of CHO cells expressing NPYR-1 or control receptors, challenged with NPY-8 or control peptides. NPY-8 specifically activates NPYR-1, while two other NPY receptors (NPYR-7 and NPYR-8) are not activated upon NPY-8 treatment. Scrambled NPY-8 was used as a negative control. Calcium responses were normalized to the total calcium response after addition of 0.1% Triton X-100. ATP, which activates an endogenous CHO receptor, was used to test the functionality of the assay. Peptides and ATP were tested at 10 and 1 μM, respectively. (C) Concentration-response curves for the activation of NPYR-1 by NPY-8 and control peptides. Data are shown as a percentage of the highest normalized response of the concentration series. NPY-8 activates NPYR-1 at EC_50_ = 36.7 nM (blue). Closely related NPY-1 fails to activate NPYR-1 (purple). Empty pcDNA3.1 vector and NPYR-7 were used as negative controls. Error bars in B and C represent standard error of the mean (SEM) (*n* ≥ 4). The underlying data for receptor assays can be found in S4 Data. (D) Colorimetric ISH showing expression of *npyr-1* in a subset of cells in the brain and along the ventral nerve cords. Inset shows the brain region at higher magnification. (E–G) Double-FISH labeling *npyr-1* (red) and other neural markers (green). *npyr-1*^*+*^ cells express neuroendocrine cell marker *pc2* (E, 28/30 express *pc2*) but not the cholinergic neuronal marker *ChAT* (F, 0/30 express *ChAT*). *npy-8* and *npyr-1* are expressed in distinct populations of cells (G, 0/30 *npyr-1* cells express *npy-8* and 0/30 *npy-8* cells express *npyr-1*). DAPI (grey) labels nuclei. Scale bars are 1 mm in D and 50 μm in E–G.

To test whether NPY-8 is able to functionally activate NPYR-1, we performed a cell-based receptor-activation assay. We individually expressed three NPY receptors encoded by the *npyr-1*, *npyr-7*, and *npyr-8* genes in CHO cells co-expressing the promiscuous Gα_16_ subunit and mitochondrially targeted apoaequorin (CHO/mtAEQ/G16) [43], which enable sensitive monitoring of intracellular calcium responses to exogenous ligands. We then assayed receptor activation after addition of various concentrations of synthetic NPY-8, as well as a closely related family member, NPY-1, and scrambled NPY-8 as controls (Fig 3A). NPYR-1 was activated by NPY-8, but not by the control ligands; by contrast, cells expressing NPYR-7 or NPYR-8, or transfected with an empty vector were not activated (Fig 3B and 3C). Moreover, concentration-response assays showed that NPY-8 activates NPYR-1 at nanomolar concentrations, with an EC_50_ value of 36.7 nM (Fig 3C). Taken together, our results suggest that NPYR-1 is the cognate receptor for NPY-8.

### *npyr-1* Is Expressed in Neuroendocrine Cells in the CNS

To identify tissues potentially targeted by NPY-8 signaling, we characterized the expression pattern of *npyr-1*. Colorimetric ISH revealed that *npyr-1* is expressed specifically in a subset of cells in the brain and ventral nerve cords (Fig 3D). We did not detect *npyr-1* expression in the gonads or accessory reproductive tissues, suggesting that NPY-8 signaling does not directly target reproductive tissues. *S*. *mediterranea* exists in two distinct biotypes: hermaphroditic sexuals that reproduce by cross-fertilization, and asexuals that reproduce by fission. Asexual planarians specify PGCs but lack differentiated germ cells and accessory reproductive tissues [44]. Interestingly, asexual planarians express *npyr-1* at levels slightly higher than sexuals (S3D Fig). Asexuals, however, express *npy-8* at ~50-fold lower levels, likely not enough to activate the *npyr-1* receptor (S3D Fig).

To identify the *npyr-1*^*+*^ cells in the CNS, we examined coexpression of *npyr-1* and two other nervous system markers: *Smed-prohormone convertase 2* (*pc2*, peptidergic neural cells) [45] and *Smed-choline acetyltransferase* (*ChAT*, cholinergic neurons) [46]. Fluorescent in situ hybridization (FISH) indicated that most *npyr-1*^*+*^ cells are *pc2*^*+*^*/ChAT*^*-*^, suggesting that these cells are not cholinergic neurons, but rather neuroendocrine cells that express and release other neuropeptides or hormones (Fig 3E and 3F). Moreover, *npyr-1*^*+*^ cells do not express *npy-8*, inconsistent with an autocrine NPY-8 signaling loop (Fig 3G). Together, our results suggest that NPY-8 targets CNS peptidergic cells through the *npyr-1* receptor, resulting in downstream signaling that eventually regulates germ cell maturation. Although the identity of the signal(s) mediating communication between the CNS and reproductive system remains unknown, the latter must have the capacity to receive and interpret such cues to regulate reproductive output.

### A Subset of Planarian GPCRs Is Enriched in Reproductive Tissues

**Fig 4:**
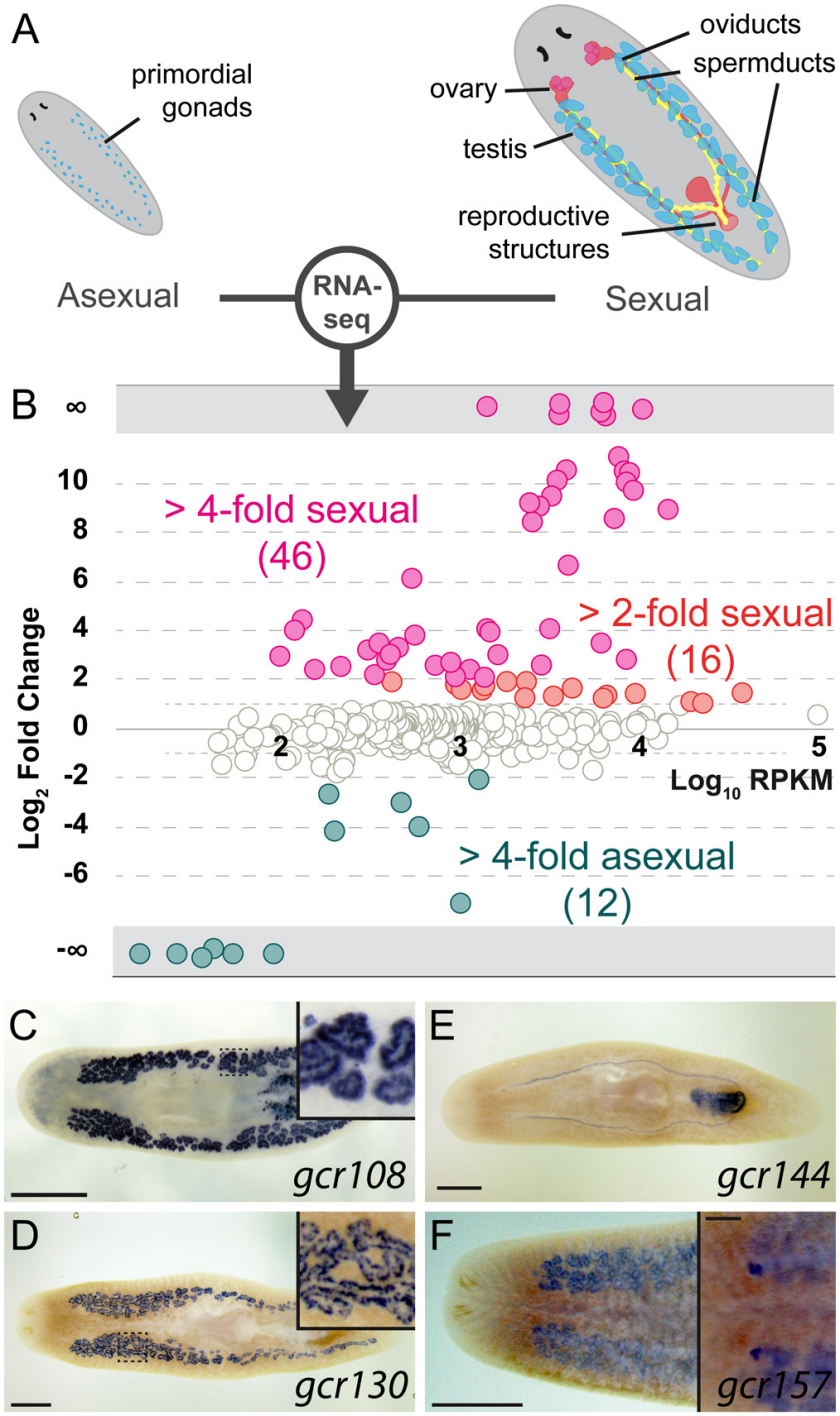
A subset of planarian GPCRs is enriched in reproductive tissues. (A) Schematic of the reproductive system in the two biotypes of *S*. *mediterranea*. Sexual planarians (right) develop a complete reproductive system, including mature gonads and accessory reproductive organs. The asexuals (left) contain only presumptive gonads with PGCs. (B) Normalized RNA-seq RPKM ratios between sexual and asexual planarians plotted against relative abundance of each GPCR gene. Only data points with *p*-value < 0.05 are shown. (C–F) Representative colorimetric ISH experiments used to validate RNA-seq results (*n* = 24/27 genes tested expressed in sexual organs). Sexually enriched genes are expressed in various reproductive tissues, including spermatids (C), spermatogonia (D and F), oviducts and female copulatory apparatus (E), and ovaries (F). Scale bars are 1 mm. Insets in C and D show the area inside the dashed box. Inset in F shows the ventral side and the scale bar is 200 μm. See also S4 Fig and S3 Data.

Because GPCRs are the largest group of cell-surface receptors, we expected to identify additional GPCRs expressed in the reproductive tissues, enabling responses to local or systemic cues. To select such candidate genes, we compared transcriptomes of the *S*. *mediterranea* sexual and asexual biotypes (Fig 4A). Genes enriched in the germline and reproductive tissues account for the majority of the differences between transcriptomes of the two biotypes [47].

Of 566 GPCRs, 46 (~8%) are up-regulated in sexual planarians (≥4-fold and *p*-value < 0.05, Fig 4B, S3 Data). In order to validate expression of candidate GPCRs in reproductive tissues we performed ISH on sexually mature planarians. The majority of sexually enriched genes (24 of the 27 tested) are expressed in reproductive tissues (S4 Fig and S3 Data). In testes, with the exception of *gcr108*, which is expressed in spermatocytes and spermatids (Fig 4C), all the other examined receptors are enriched in spermatogonial cells (e.g., *gcr130*, Fig 4D). Other GPCRs are expressed in accessory reproductive organs, such as oviducts and copulatory apparatus (e.g., *gcr144*, Fig 4E). Only one of the tested GPCRs, *gcr157*, is enriched in both female and male germ cells (Fig 4F). These results implicate GPCRs in reception of signals by germ cells and their associated somatic tissues.

### The Orphan Receptor *ophis* Is Required for Germ Cell Differentiation and Reproductive Maturity

**Fig 5:**
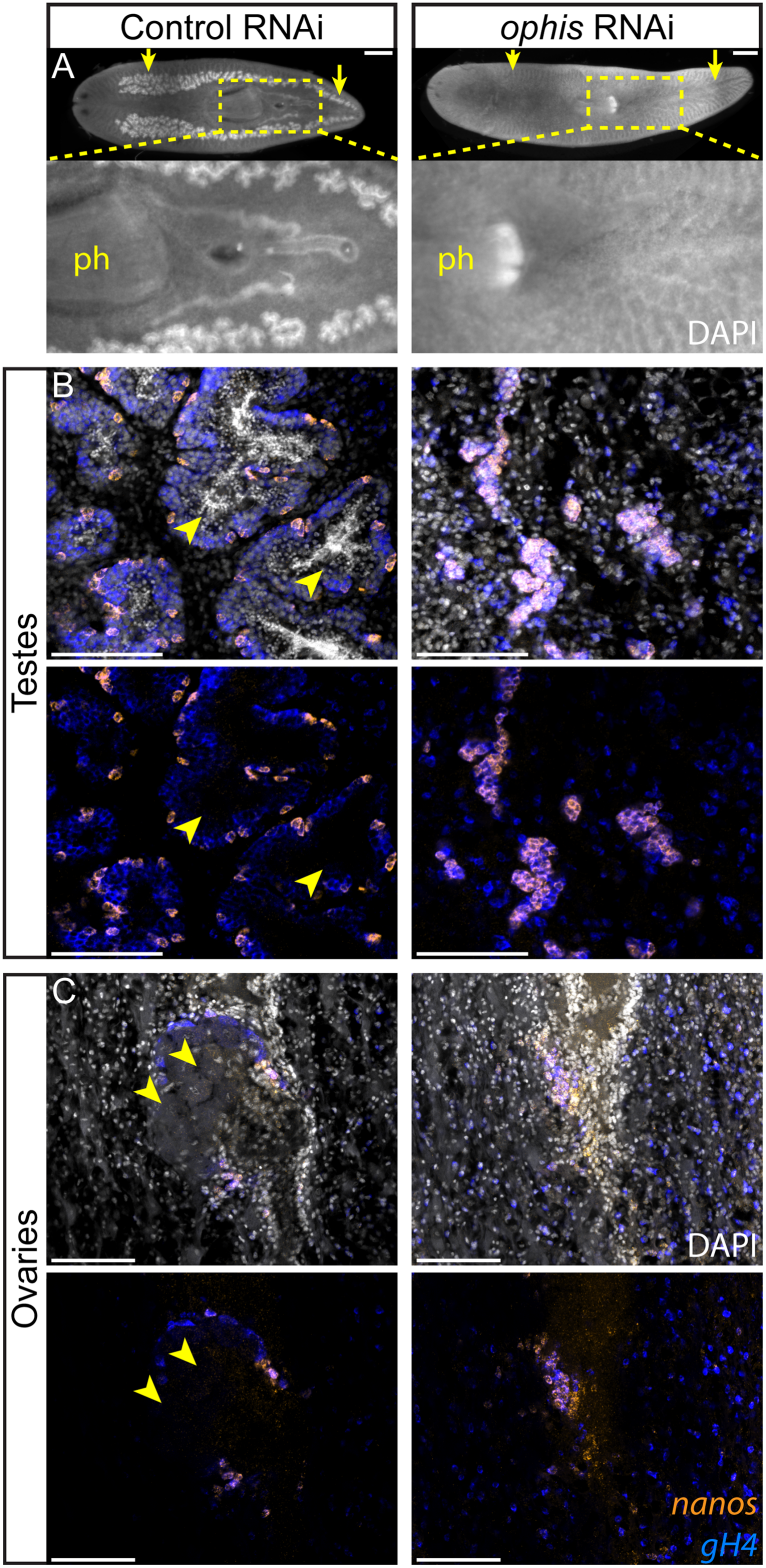
*ophis* is required for male and female germ cell differentiation. (A) Whole-mount DAPI staining of control and *ophis(RNAi)* worms shows testes (yellow arrows) and stored sperm (inset). Control animals (*n* = 5/5) possess a mature reproductive system, including all differentiated cell types of the testes and ovaries, *ophis(RNAi)* worms (*n* = 15/15, three independent experiments) lack gonads and mature gametes. RNAi treatment (8 dsRNA feedings) started in hatchlings (see Fig 2A for the dsRNA feeding paradigm). RNAi quantification data can be found in S4 Data. (B and C) Double-FISH labeling GSCs (*nanos*^*+*^, orange) and undifferentiated germ cells (*nanos*^*-*^*/gH4*^*+*^, blue) in control and *ophis(RNAi)* worms. In testes (B), *ophis(RNAi)* worms contain only *nanos*^*+*^ GSCs and are devoid of *nanos*^*-*^*/gH4*^*+*^ spermatogonial cells and DAPI-rich spermatids and sperm. Control worms have fully developed testes with spermatids and sperm in the middle of lobes (arrowheads). In ovaries (C), mature oocytes are observed in control animals (arrowheads) but not in *ophis(RNAi)* worms. See Fig 2B for a schematic representation of the spatial organization of the gonads. DAPI (grey) labels nuclei. Scale bars are 1 mm in A and 100 μm in B and C.

To determine whether any of the sexually enriched GPCRs are required for reproductive development, we performed an RNAi screen starting with planarian hatchlings (Fig 2A). We found that knockdown of a serpentine receptor family (*Srf/w*) member we named *“ophis”* (after the mythological serpent wrapping around the Orphic Egg), resulted in animals with immature testes that lack differentiating *gH4*^*+*^ spermatogonial cells, spermatocytes, spermatids, and sperm (Fig 5A and 5B, S4 Data). Ovaries were also affected, revealed by the absence of mature oocytes (Fig 5C, S4 Data). Seminal vesicles with stored sperm were not observed in *ophis(RNAi)* worms (Fig 5A). Despite the loss of all differentiating germ cells, all *ophis(RNAi)* animals retained their pool of *nanos*^*+*^ GSCs (Fig 5B and 5C). Therefore, *ophis* is required for differentiation, but not maintenance, of *nanos*^*+*^ GSCs.

### *ophis* Is Expressed in Somatic Reproductive Tissues, Including Gonadal Niche Cells

**Fig 6:**
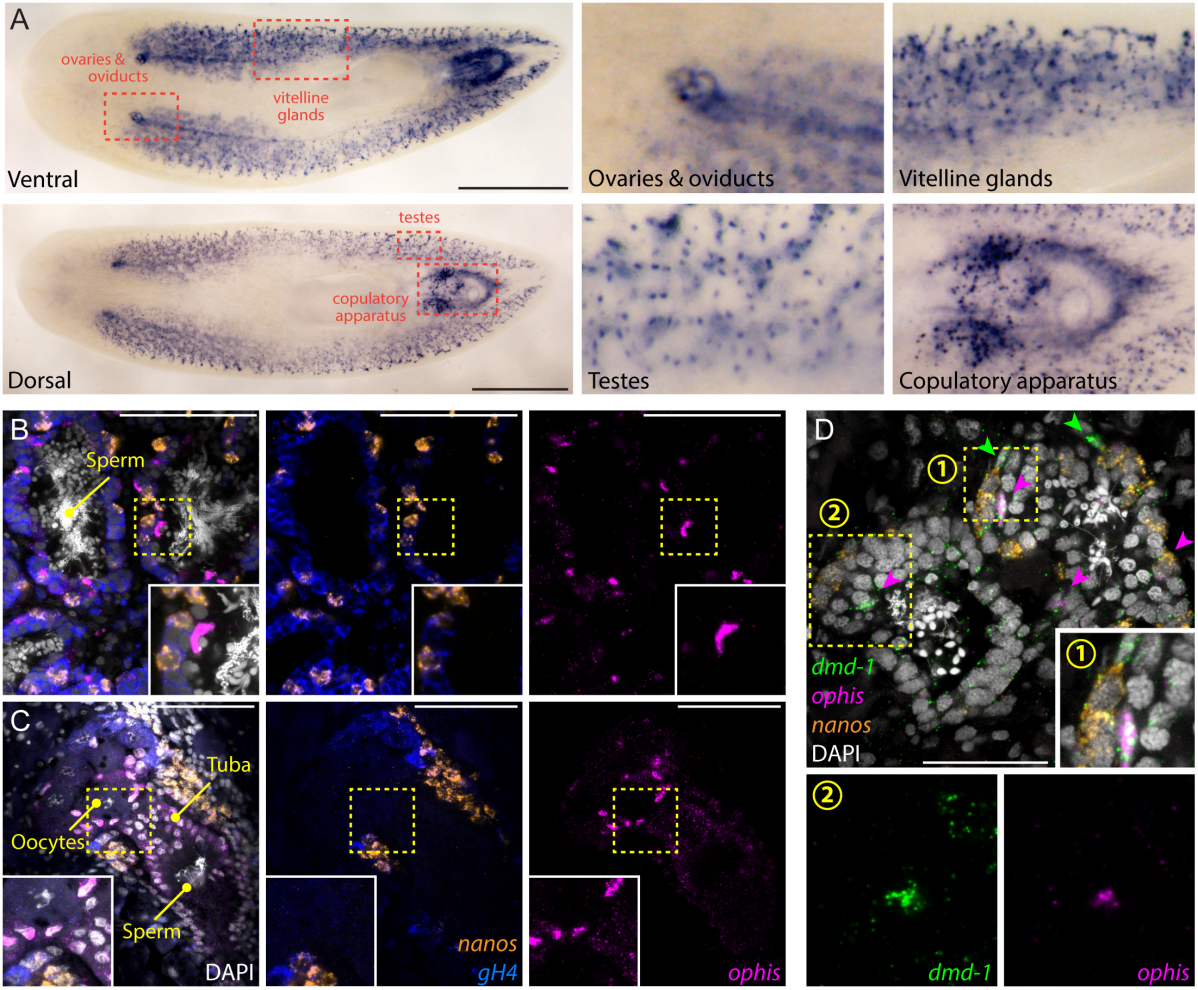
*ophis* is expressed in the somatic gonadal niche. (A) Colorimetric ISH shows expression of *ophis* in somatic reproductive structures. Insets show magnified view of specific tissues indicated by red dashed boxes. (B and C) Triple-FISH labeling *ophis* (magenta), *gH4* (blue), and *nanos* (orange). Within gonads, *ophis* expression is exclusive to somatic cells in the periphery of testis lobes (B) and in presumptive follicular cells of the ovaries (C). (D) Triple-FISH labeling *ophis* (magenta), *nanos* (orange), and *dmd-1* (male somatic gonad cells, green) in the testes. *ophis* and *dmd-1* are co-expressed inside testes (magenta arrowheads). *dmd-1*^*+*^*/ophis*^*-*^ cells can be seen outside the testes (green arrowheads). Insets 1 and 2 show magnification of regions indicated by numbered yellow dashed boxes. DAPI (grey) labels nuclei in B–D. Scale bars are 1 mm in A, 100 μm in B and C, and 50 μm in D.

To determine where *ophis* is expressed in sexual planarians, we performed whole-mount ISH. We detected *ophis* expression in several accessory reproductive tissues, including oviducts, tuba, vitellaria, and parts of the copulatory apparatus, as well as in discrete cells in testes (Fig 6A). To characterize the cell types in which *ophis* is expressed in the gonads, we performed FISH. In both testes and ovaries, *ophis* is detected in somatic cells (*nanos*^*-*^*/gH4*^*-*^) closely associated with germ cells (Fig 6B and 6C). We previously showed that a few somatic cells within each testis lobe express a conserved sex-specific transcription factor (*Smed-dmd-1*) and lack known germline markers [48]. These *dmd-1*^+^ cells are required for specification and maintenance of *nanos*^*+*^ GSCs and are thought to contribute to a presumptive germline niche. FISH experiments revealed coexpression of *ophis* and *dmd-1* transcripts in the somatic cells of the testes (Fig 6D). The nuclei of these cells have an elongated and angular shape, distinct from the round nuclear morphology of spermatogonia and spermatids. Furthermore, somatic cells of the testes seem to have an expanded cytoplasm, delineated by *dmd-1*^*+*^ puncta that stretch between germ cells (Fig 6D).

A population of *dmd-1*^*+*^ cells also exists in the dorsal mesenchyme (between testis lobes) and are potential progenitors of somatic gonadal cells [48]. By FISH, these cells seem to express higher levels of *dmd-1* compared to the somatic gonadal cells and do not express *ophis* (Fig 6D). On the ventral side, *ophis* is expressed abundantly in vitellaria, copulatory organs, oviducts, and ovaries (Fig 6A and 6C). In oviducts and ovaries, expression of *ophis* resembles that of *Smed-nhr-1*, a nuclear hormone receptor required for planarian germ cell development [49]. *ophis*^*+*^ cells in the ovary are also *nanos*^*-*^*/gH4*^*-*^, suggesting that they represent somatic cells of planarian ovaries (Fig 6C).

### *ophis* Knockdown Does Not Affect Male GSC Specification

**Fig 7:**
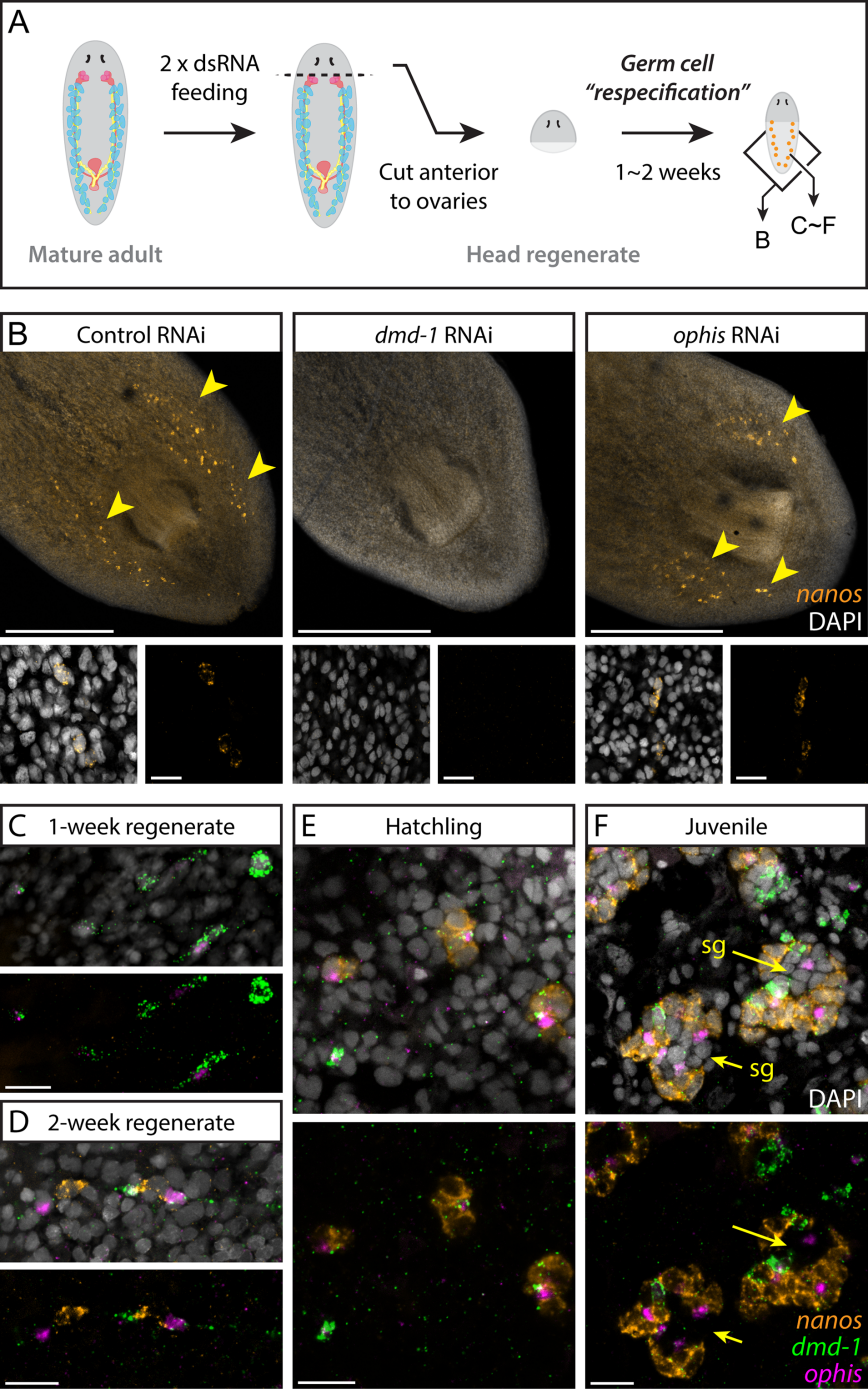
Somatic *dmd-1*^*+*^*/ophis*^*+*^ cells facilitate testis regeneration and development in planarians. (A) Germ cell re-specification paradigm used to challenge worms to specify a germline de novo. Indicated genes were knocked down in worms prior to amputation. After 1 to 2 wk of posterior regeneration, regenerates were fixed and labeled to detect *nanos* expression. Schematic shows areas imaged in panel B and panels C–F. (B) FISH labels *nanos*^*+*^ GSCs in 2-wk head regenerates of control, *dmd-1(RNAi)*, and *ophis(RNAi)* planarians. Unlike *dmd-1*, *ophis* is not required for de novo germ cell specification during regeneration of head fragments. Insets show early *nanos*^*+*^ GSCs. (C-F) FISH showing expression of *dmd-1*, *ophis*, and *nanos* during de novo gonad regeneration. At one week post-amputation (C), *dmd-1*^*+*^*/ophis*^*-*^ and *dmd1*^*+*^*/ophis*^*+*^ cells are detected at the posterior half of head regenerates. Most worms are devoid of *nanos*^*+*^ germ cells (*n* = 8/10). At 2 wk (D), *nanos*^*+*^ cell clusters appear adjacent to *dmd-1*^*+*^*/ophis*^*+*^ cells (*n* = 10/10). Early hatchlings (<2 wk old, E) express clusters of *nanos*^*+*^ cells near *dmd-1*^*+*^*/ophis*^*+*^ somatic cells. No differentiated germ cells (*nanos*^*-*^) are observable within the clusters. In juveniles (F), in addition to all of the previous combinations, testis lobes with more differentiated spermatogonial cells (“sg” and arrows, *nanos*^*-*^) appear in the middle of the clusters. Quantification of the observations in C–F can be found in S4 Data. DAPI (grey) labels nuclei. Scale bars are 500 μm in B and 20 μm in insets and C–F. See also S5 Fig.

Since *ophis* RNAi did not affect the maintenance of *nanos*^*+*^ GSCs, we tested whether initial specification of GSCs required somatic *ophis* expression. We analyzed *ophis(RNAi)* worms through the de novo GSC re-specification paradigm shown in Fig 7A using *dmd-1(RNAi)* worms as positive controls. While *dmd-1(RNAi)* worms failed to re-specify *nanos*^*+*^ PGCs, re-specification appeared normal in control and *ophis* knockdown worms (Fig 7B). Our results indicate that although *dmd-1* and *ophis* are both expressed in the somatic testis cells, their functions differ in that *ophis* knockdown does not affect specification of GSCs or their maintenance.

### A Subset of *dmd-1*^*+*^ Cells Expresses *ophis* and Establishes New Testes

The appearance of *nanos*^*+*^ GSCs has been the earliest known event marking the establishment of new testes in planarians, and previous work on *dmd-1* did not directly address the role of the somatic gonadal cells in early gonadogenesis. With identification of *ophis* as a second marker for the somatic gonadal cells, we re-examined the developmental events leading to formation of a new testis. With more sensitive ISH techniques [50] we find that the majority of sexual planarians possess *nanos*^*+*^ cells at hatching. To recapitulate the earliest stages of gonadogenesis (before expression of *nanos* in PGCs) we forced the worms to specify new gonads de novo. To this end, we allowed head fragments from wild-type planarians to regenerate new tails and simultaneously monitored expression of *dmd-1*, *ophis*, and *nanos* by FISH in the regenerated tissues. We observed that *nanos*^*+*^ cells are rarely present one week after amputation, however somatic *dmd-1*^*+*^*/ophis*^*-*^ and *dmd-1*^*+*^*/ophis*^*+*^ cells appear dorsally (Fig 7C). After 2 wk of regeneration, *nanos*^*+*^ GSCs are present adjacent to the *dmd-1*^*+*^*/ophis*^*+*^ cells on the dorsal side (Fig 7D). Notably, no *nanos*^*+*^ cells can be found isolated from presumptive somatic testis cells, suggesting that direct contact with the somatic niche is required for GSC specification and maintenance.

We also followed the progression of gonadogenesis by performing FISH on planarian hatchlings. Early hatchlings (<2 wk) resemble 2-wk head regenerates in that they possess *dmd-1*^*+*^ and *dmd-1*^*+*^*/ophis*^*+*^ cells as well as *nanos*^*+*^ GSCs (Fig 7E). In juvenile planarians (>2 wk), primitive gonads containing differentiating *nanos*^*-*^ germ cells can be observed (Fig 7F). In regenerating head fragments, early hatchlings, and juveniles, *dmd-1*^*+*^*/ophis*^*+*^ cells can be found outside of presumptive testes (in the mesenchyme, Fig 7C–7F), which is not the case with adult planarians (i.e., *ophis* expression can only be detected in somatic cells within the gonads of adult planarians) (Fig 6D, sheet “Fig 7C–7F” in S4 Data). This suggests that during homeostasis, *dmd-1*^*+*^ cells either join pre-existing testis lobes before expressing *ophis*, or that de novo testis formation around a *dmd-1*^*+*^/*ophis*^*+*^ cell occurs very rapidly. Asexual planarians, which only specify GSCs but are unable to produce gametes, possess *dmd-1*^*+*^ gonadal niche cells associated with clusters of *nanos*^*+*^ GSCs (S5A Fig). Consistent with the RNA-seq data, *ophis* expression is lower in asexual worms (~5-fold) and cannot be detected by FISH (S5A and S5B Fig). These results support the hypothesis that GSCs can give rise to differentiated germ cells only in association with somatic gonadal cells that express *ophis* at detectable levels.

## Discussion

We investigated the function of planarian GPCRs in different aspects of germline function. We performed a comprehensive bioinformatics analysis to identify and classify GPCRs of the planarian *S*. *mediterranea*, followed by expression and functional studies to characterize roles for these genes in reproductive development. We identified homologs of the NPY receptor family and showed that the CNS-expressed *npyr-1* is required for differentiation of germ cells into mature gametes in a manner similar to that of the previously identified NPY-like peptide, *npy-8* [32]. By in vitro receptor assays, we demonstrated that synthetic NPY-8 can specifically activate NPYR-1. Next, to identify receptors that act to regulate the reproductive tissues, we focused on GPCRs enriched in the sexual strain of *S*. *mediterranea*. We found that genes in this category are mainly associated with the germline and somatic reproductive tissues. One sexually enriched gene, *ophis*, is expressed in somatic gonadal niche cells (among other reproductive tissues) and is required for differentiation of both male and female GSCs. We also found that, in testes, *ophis* is co-expressed with *dmd-1*. However, unlike *dmd-1*, *ophis* is not essential for GSC specification or maintenance. Therefore, the *ophis* phenotype uncovers a secondary role for somatic gonadal cells in supporting GSC differentiation.

### Novel Subfamilies of Rhodopsin-Like GPCRs Have Evolved in Flatworms

Due to the near-completeness of the genome and abundance of high-quality transcriptomic data, our bioinformatics analysis has likely identified the full complement of planarian GPCRs. This collection has increased the number of known planarian GPCRs from 343 to 566, and significantly improved the average number of discovered transmembrane (TM) domains to over 6.8. Using a combination of similarity-based clustering and phylogenetic methods, we were able to classify 516 GPCR genes (91%) into five conserved GRAFS families; contrary to a previous report [26], no significant clusters of non-GRAFS GPCRs were identified in the planarian genome.

The rhodopsin family of GPCRs is remarkably expanded in planarians. Only 143 out of 461 of planarian rhodopsin-like GPCRs (*Rho-C*) cluster with vertebrate counterparts and the rest form divergent subfamilies *Srf/w*, *D*, *L*, and *R*. *Srf/w* includes representatives of the *srw* family of chemoreceptors. Our data support the hypothesis that the invertebrate chemoreceptor family split from the peptide subfamily of receptors sometime around the divergence of the protostome ancestor [51]. Other chemoreceptor-like genes identified in this study (*Srfa*, *Srfb*, and *Srfc*) as well as *Rho-L*, *D*, and *N* have no previously reported homologous families, rendering them as potential flatworm-specific groups. *S*. *mediterranea* is among the most experimentally tractable members of the lophotrochozoan superphylum, the biology of which is relatively unexplored compared to vertebrates or ecdysozoans. Study of a potentially vast number of functionalities (e.g., neurotransmission, pheromone signaling, structural roles) facilitated by the GPCR subfamilies discovered in this work will enrich our understanding of the diversity of strategies utilized in metazoans development and physiology.

### Central NPY Signaling May Have a Conserved Role in Reproductive Development

Previous studies in mammals and *Drosophila* have failed to depict a clear and consistent picture of how NPY and its receptors are involved in reproductive function. NPF expression in *Drosophila* brain is sexually dimorphic and is believed to be centrally involved in mating behavior [52]. Also, NPF-deficient flies show a decrease in egg laying capacity, but the same effect is not observed in NPFR-1-deficient flies [39]. In mammals, injection of NPY into sex steroid-primed ovariectomized rats induces secretion of LH and GnRH [53,54]. Conversely, in intact rats, NPY has an inhibitory effect on reproduction by suppressing the pituitary-gonadal axis [55]. This effect is exacerbated under conditions of negative energy balance, when the endogenous hypothalamic NPY levels are high, suggesting that NPY is responsible for coordinating reproductive function with energy availability [56].

NPY receptors are abundant in the CNS of animals, with NPY1R and NPY2R being highly enriched in mammalian brains, and NPFR1 expressed in a small number of *Drosophila* brain cells, suggesting a conserved central role for NPY signaling in regulation of physiological functions [39,57,58]. Deletion of Y1 and Y4 receptors in mice can, under some conditions, enhance GnRH, LH, or sex hormone levels, or mammary gland development, suggesting that NPY receptors may act to limit aspects of reproductive development [59,60]. In contrast, NPY positively modulates GnRH neuronal output in mammals and teleosts [61,62], implying a systemic pro-germline regulatory function for NPY. Further complicating the picture, deletion of NPY or its receptors in many other studies has led to no obvious changes in reproductive function, presumably due to functional redundancy (among NPY and its paralogs or the NPY receptors) or compensatory mechanisms [63,64].

**Fig 8:**
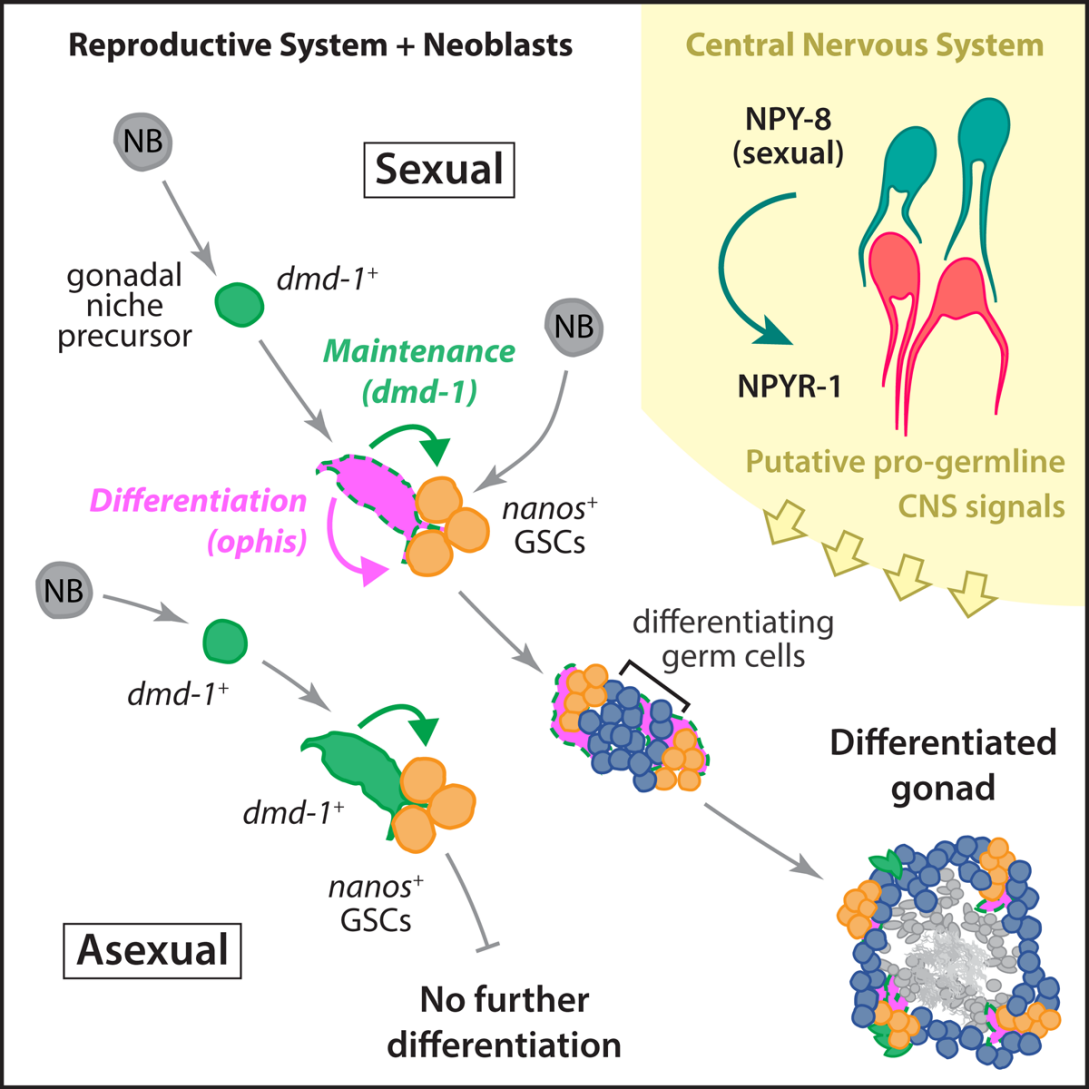
Schematic of the developmental mechanisms involved in planarian testis formation. *dmd-1*^*+*^ cells in both sexual and asexual worms are required for specification of *nanos*^*+*^ GSCs. In sexual planarians, these *dmd-1*^*+*^ cells express *ophis*, which is required for further differentiation of GSCs into mature gametes. NPY-8 signaling, which occurs in the CNS, systemically promotes later stages of germ cell maturation.

Our studies have shown that neurally expressed NPY-8 and its receptor within the CNS, NPYR-1, are required for proper germline development in planarians (Fig 8). This is consistent with, and may help explain the regression of the reproductive system observed upon head amputation in planarians [19,20]. Our findings suggest that NPY signaling plays a conserved role in regulation of reproductive development and expression of the *npyr-1* receptor in the planarian CNS makes for an even more compelling case of evolutionary conservation of NPY signaling function. Furthermore, the NPY receptor identified in this work, *npyr-1*, provides an entry point for cellular and molecular studies of NPY receptor signaling and its downstream pathways and binding partners.

### Diversity of Planarian Rhodopsin Family Reveals Novel Insights into Invertebrate Chemoreception

We show that the large metazoan rhodopsin family has split into multiple diverse subfamilies in planarians, of which, only one (*Srw*) has been reported in other animals. Much like in nematodes, planarian GPCRs can potentially act as a versatile toolbox enabling them to respond to a wide range of molecules and ligands associated with food, predators, or mates [65]. Chemosensation through sensory neurons can coordinate germ cell development with population size and food abundance in *C*. *elegans* [66]. However, *ophis* represents an interesting case in which a chemoreceptor family member expressed within the somatic niche, rather than specialized sensory neurons, regulates germ cell development. No direct homologs of *ophis* have been identified in other organisms so far and characterization of its ligand, mechanism of action, and downstream pathways will require further studies. Although a majority of invertebrate chemoreceptors are as yet orphan, some have been directly linked to odorant or pheromone sensation with developmental consequences [37,67]. Whether *ophis* responds to a pheromone-like molecule, a systemic hormone, or a feedback signal from the developing germ cells remains to be discovered. The receptor-activation assay used in this work can be used in conjunction with biochemical fractionation methods to de-orphanize *ophis* and other planarian chemoreceptors.

### A Dual Role for Planarian Somatic Gonadal Niche Cells

GSCs are dependent on interactions with specialized somatic cells to maintain their identity and proper function [68]. In the mammalian testis, the environment created in the vicinity of blood vessels by myoid cells, the basement membrane, and specialized domains within Sertoli cells provides the niche for male GSCs [69]. The expansive cytoplasm of Sertoli cells also surrounds germ cells and supports their differentiation throughout spermatogenesis [70]. However, in *Drosophila* and *C*. *elegans*, there is a division of labor among somatic cells of the gonad, with cap/hub cells and distal tip cells providing the niche for GSCs and escort/cyst/follicle cells and sheath cells supporting differentiation of germ cells [71–73]. In the planarian testes, the *dmd-1*^*+*^*/ophis*^*+*^ cells seem to perform both functions simultaneously, much like the Sertoli cells of mammalian testes (Fig 8). It is conceivable that somatic ovarian cells are responsible for both the maintenance and differentiation of female germ cells as well; however, no genes required for the former function have yet been identified in planarians.

### Planarian GPCR Research Can Guide Similar Studies in Parasitic Flatworms

Parasitic members of the phylum Platyhelminthes are equally impressive with regards to their reproductive development, managing to maintain reproductive potential throughout the many stages of their life cycle and reaching sexual maturity at the appropriate time and place within the suitable host [74,75]. Furthermore, parasitic flatworms infect most vertebrate species, causing major health concerns in developing countries [76]. Currently, praziquantel is the only therapeutic agent available for schistosomiasis treatment [77], and the threat of developing resistance in patients warrants a systematic search for novel therapeutic targets. Characterization of the planarian GPCR complement will guide similar studies in related parasitic flatworms such as *Schistosoma mansoni* in which flatworm-specific receptor families could constitute potential drug targets [26].

### Conclusions

Our analyses characterized the complement of planarian GPCRs and pave the way for studying how this major group of cell-surface receptors is involved in developmental and physiological functions, such as reproduction, regeneration, organogenesis, chemosensation, and adaptation. In this work, we examined two cases in which GPCRs are involved in regulation of the germline: central NPY signaling that is required for sexual maturation, and a niche-level mechanism in which a novel chemoreceptor family member promotes germ cell differentiation. In a broader context, this work lays the groundwork for future characterization of parasitic flatworm GPCRs for basic biology and drug discovery purposes, and provides insights into the diversity and evolutionary history of metazoan GPCRs.

## Materials and Methods

### Planarian Husbandry

Sexual and asexual *S*. *mediterranea* were maintained at 18°C in 0.75X Montjuïc salts [78] or 0.5 g/L Instant Ocean Sea Salts (Spectrum Brands), respectively. Animals were starved at least one week prior to use. For all experiments with sexual *S*. *mediterranea*, worms ≥8 mm length were used, unless otherwise specified.

### Discovery of Planarian GPCRs

To maximize coverage of the sexual and asexual strains of the planarian, we developed two strain-specific de novo transcriptomes which we then combined and used for receptor mining (FASTA file in S1 Data). For some gene curations, we also used a publicly available assembly that was generated through a different methodology (PlanMine v2.0beta, http://planmine.mpi-cbg.de/planmine/). Transcriptomes were mined using tBLASTx (BLOSUM62) and HMMer [79] to identify potential receptor sequences. Conserved GPCRs from other organisms and previously published planarian GPCR sequences [26] were used as a seed dataset to discover planarian GPCRs. Where possible, we expanded and curated the transcripts to reach the ends of the open reading frames (ORFs). ORFs were marked “N-term OK” if we found a stop codon closely followed by an AUG start codon at the 5′-end of the transcript. ORFs were marked “C-term OK” if we found a stop codon at the 3′-end. If both of these conditions were met, the entry was marked “ORF confident.” A pool of potential receptor sequences was then filtered to exclude non-GPCR sequences, namely, those that showed significant similarity to other types of transmembrane proteins, such as ion channels or solute carrier proteins (E-value < 1E-10 for at least half of the top-fifty tBLASTx hits against NCBI nucleotide collection). We also removed genes that appeared to have a complete ORF but encoded a protein that did not show the correct topology: seven TM domains flanked by an extracellular N-terminus and an intracellular C-terminus (determined by TMHMM2.0) [80]. To complement the topologies calculated by TMHMM2.0, we inspected the posterior probability graphs generated by the program looking for potential TM domains that did not pass the 50% threshold to be reported by the program. Such curations are noted in the table in S3 Data. After a second round of receptor mining and filtering, a set of 566 GPCR genes was finalized for downstream analyses. See S1A Fig for the workflow we followed. Additional recursive searches did not expand the number of putative receptors, suggesting that our database contains nearly all of the expressed GPCR genes and the possibility of a significant number of unidentified GPCRs is slim.

### Classification of Planarian GPCRs

Initial compartmentalization of the planarian GPCRs was done by calculating an all-against-all similarity matrix of the 566 sequences that was then reduced to three-dimensional similarity networks. Human, insect, and nematode GPCRs were also included in parallel analyses to aid with identifying functional categories (S1B Fig). BLASTP was used to generate the similarity matrix, which was then analyzed by CLANS 2.0 [33] to determine the three-dimensional or two-dimensional cluster representation. CLANS 2.0 was allowed to use similarities more significant than 1E-9 and cycle approximately 20,000 times to optimize the graph. For the cross-species clustering, the E-value threshold was set at 1E-6. The planarian GPCR graph was subsequently analyzed to extract convex clusters of four or more genes with the attraction value limit set at 0.5 standard deviation.

Next, we pooled the rhodopsin-like genes from the previous analysis and aligned them using ClustalW [81] within CLC Genomics Workbench 7. We then generated a phylogenetic tree by the neighbor-joining method (Jukes-Cantor distance measure), with 1000 replicas to deduce bootstrap values. We used this tree to validate the clustering analysis results. Two small clusters, containing 13 and five genes, were merged with clusters *Srfa* and *Srw*, respectively, because they were bound within the representatives of their parent clusters on the tree. On the other hand, cluster *R1* was extracted from cluster *Rho-C*, because its members were more closely related to those of cluster *R2* than the original cluster. Finally, *gcr081*, *gcr089*, *gcr442*, and *gcr500* were moved from *L1* to *L2* based on their positions on the phylogenetic tree.

### NPY Receptor Phylogeny

The 16 putative NPY receptor genes were used to perform an alignment search against Reference Proteomes with the EMBL-EBI HMMER tool (http://www.ebi.ac.uk/Tools/hmmer/). Representative hits with E-value < 1E-30 were aligned and phylogenetic trees were constructed. In a separate analysis, only the planarian NPY receptors and their parasitic flatworm homologs were used. ClustalW (within CLC Genomics Workbench 7.0) was used to align amino acid sequences with default parameters. The resulting alignments were subjected to phylogenetic analysis by Bayesian inference in MrBayes v.3.2 [82], using the Whelan and Goldman (WAG) evolutionary model [83] to construct a 50% majority rule tree and assign posterior probabilities to tree nodes. Markov Chain Monte Carlo (MCMC) analyses ran on two independent chains (default) for 200,000 iterations and finished with an average standard deviation of split frequencies of 0.01 or less. The first 25% of sampled trees were discarded as the burnin period. Phylogenetic trees were visualized by FigTree v.1.4 (http://tree.bio.ed.ac.uk/software/figtree/). All trees were rooted with a group of two human and two planarian amine receptors.

### Receptor Cloning

For the purpose of ISH and RNAi experiments, 500–1,000 bp segments of the GPCR genes were amplified (primers in S5 Data) using Platinum Taq DNA Polymerase (Invitrogen). Products were TA-cloned into pJC53.2 and sequenced to determine the directionality of cloning. pJC53.2 allows for probe synthesis using SP6 or T3 RNA polymerases, as well as dsRNA synthesis using T7 RNA polymerase [32].

A number of full-length NPY receptor coding sequences were cloned into pcDNA3.1 for the purpose of in vitro receptor assays. Primers corresponding to *npyr-1* (forward: ACAGGGATCCACCATGGATTTGTGTAAGGATAATC, reverse: TATAGAATTCAGGACGACGATACTTCACTTTTG), *npyr-7* (forward: ACAGGGATCCACCATGAATTCTATGAAAAATC, reverse: TATAGAATTCATAAAGATGATATTTTGAATCTTC), *npyr-8* (forward: ACAGGGATCCACCATGATTTTATCGAATGGC, reverse: TATAGAATTCAATTTACTAATCCAATATGAGAATC), *ophis* (forward: ACAGGGATCCACCATGGTTTTCTGTAGACTAAT, reverse: TATAGAATTCAATTTATCGTTGAAGATTG) were used to amplify from sexual planarian cDNA. Forward primers had an extension containing a *BamHI* site and a Kozak sequence before the start codon, while reverse primers contained a stop codon followed by an *EcoRI* site. Resulting clones were sequenced from both ends to confirm completeness and directionality of the insert. Plasmids were purified by QIAGEN Plasmid Midi Kit before transfecting CHO cells.

### RNAi Knockdown

Knockdowns were generated by feeding in vitro transcribed dsRNA, as previously described [32], and using dsRNA matching the *ccdB* and *camR*-containing insert of pJC53.2 as a control. Worms were fed with 5 μg of dsRNA combined with 45 μL of a 3:1 calf liver:water mixture. For the post-embryonic development paradigm, cut and regenerated worms smaller than 8 mm were fed dsRNA eight times, every 6 d (Fig 2A). For the re-specification paradigm, mature worms were fed two doses of dsRNA, cut anterior to the ovaries, and the head fragments were allowed to regenerate for 2 wk, unless otherwise specified (Fig 7A).

### Peptide Synthesis

NPY-1 (LNEYFAIVGRPRF-amide), NPY-8 (PMFDSADAFRNYLRKLNNEYMIAGRPRF-amide), and scrambled NPY-8 (LFRMRFDAMKDELRANNNRYFIPSYPGA-amide) were synthesized by New England Peptide (Gardner, MA). Peptides were designed and C-terminally amidated based on in silico prediction of their bioactive form after enzymatic processing [32]. For each peptide, purity of >95% was confirmed through HPLC by the vendor. Peptides were soluble in pure water (aided by brief sonication if needed).

### In Vitro Receptor-Activation Assay

In vitro receptor-activation assays were done as previously described [84]. Briefly, calcium responses were measured in CHO-K1 cells transiently transfected with a receptor::pcDNA3.1 construct of interest. Cells also stably expressed the promiscuous Gα_16_ protein and mitochondrially targeted apoaequorin. After loading with the cofactor coelenterazine, transfected cells were challenged with synthetic peptides (>95% purity) and calcium responses were simultaneously monitored on a Mithras LB940 luminometer. In each case, calcium responses evoked by peptides were normalized to the total calcium response (i.e., response evoked by the peptide plus response evoked by a second addition of 0.1% Triton X-100). For concentration-response curves, the normalized calcium responses are plotted as a percentage of the highest normalized response of the concentration series. Data were averaged from at least two independent experiments. Dose-response curves were constructed with a nonlinear regression analysis using a sigmoidal dose-response equation in Sigmaplot 12.0.

### In Situ Hybridization

Whole-mount ISH was performed with a formaldehyde-based fixation procedure as previously described [50]. The protocol was optimized for larger sexual planarians: formaldehyde fixation was increased to 30 min, proteinase K treatment was increased to 30 min, and post-fixation was increased to 20 min. Colorimetric and FISH samples were imaged on an Axio Zoom.V16 and a Zeiss LSM 710 confocal microscope (Carl Zeiss, Germany), respectively. ISH probes were synthesized according to the methods previously described [50]. Single-stranded RNA probes for *Smed-gH4* (GB: DN306099) and *Smed-npy-8* (GB: BK007010) were labeled with fluorescein isothiocyanate (FITC), for *Smed-nanos* (GB: EF035555) and *Smed-npyr-1* with dinitrophenol (DNP), and for *Smed-pc2* (GB: BK007043), *Smed-ChAT* (PlanMine, *dd_Smed_v6_6208*), and all of the analyzed GPCR genes with digoxigenin (DIG). For some FISH experiments, FITC or DNP probes for *npyr-1* and *ophis* were synthesized. For FISH experiments, probes were detected by corresponding HRP-conjugated antibodies and developed with 5-carboxytetramethylrhodamine (5-TAMRA), fluorescein amidite (FAM), or DyLight 633. For colorimetric ISH experiments, DIG-labelled probes were detected by AP-conjugated antibodies and developed with nitro-blue tetrazolium (NBT) and 5-bromo-4-chloro-3'-indolyphosphate (BCIP).

### RNA-seq Expression Analyses

Approximately 400,000 reads from 12 independent control samples (six sexual and six asexual worms; S6 Data) were mapped to the GPCR database using 0.9 as minimum similarity and coverage fractions. Base 2 logarithm of the RPKM values [85] were used as a relative measure of expression comparing the two strains. False Discovery Rate-corrected *p*-values [86] smaller than 0.05 were considered significant. Read mapping and statistical analyses were performed using CLC Genomics Workbench 7. Because sexual planarians express sexually enriched GPCRs at very high levels compared to asexuals (increasing the denominator in the RPKM fraction), other GPCRs falsely appear to be enriched in asexuals. To correct this bias, we graphed log_2_(fold change) against log_10_(RPKM) for the 376 GPCRs with significant *p*-values. We then calculated a linear regression trend line for the middle 282 (75%) data points and used it to transform all fold change values so that the majority of them are around zero. The following transformation was applied: normalized log_2_(fold change) = original log_2_(fold change) − (0.411 * log_10_(RPKM)) + 2.29.

### Quantitative PCR

Total RNA was extracted from whole individual worms using TRIzol Reagent (Invitrogen) according to the manufacturer’s instructions, using the high-salt step for RNA precipitation. RNA was DNAse-treated, purified, and concentrated using the DNA-free RNA kit (Zymo Research). About 1 μg total RNA was used to prepare cDNA using iScript cDNA Synthesis Kit (Biorad) according to the kit protocol. GoTaq qPCR Master Mix (Promega) was used for qPCR reactions in a StepOnePlus Real-Time PCR machine (Applied Biosystems). *Smed-beta-tubulin* was used as an internal control. Primers corresponding to *npy-8* (forward: TGACTCAGCTGATGCCTTTC, reverse: GCCAAATCTTGGTCTTCC), *npyr-1* (forward: ACGACATTCAACGACAGAGG, reverse: GTAACGACATCGGACCAACA), *nanos* (CAAGGACAAATGTTGCCTGTA, reverse: CAACCCATCGATCCAACTCT), *beta-tubulin* (forward: TGGCTGCTTGTGATCCAAGA, reverse: AAATTGCCGCAACAGTCAAATA), and *ophis* (forward: ATCGTCTATTGGCCCGTAAG, reverse: AAACGACTGAGCGGAACAAC) were used. Three technical replicates were assayed for each sample. At least four individual animals were used as biological replicates for each condition tested, unless mentioned otherwise. For each gene, ΔC_t_ was calculated as the difference between C_t_ values of the gene of interest and *beta-tubulin*. Error bars indicate the range of relative quantities calculated from ΔΔC_t_ ± SEM, where SEM is the standard error of the mean of ΔC_t_ values of the test (and not the reference) biological replicates.

## Supporting Information

S1 Data *S*. *mediterranea* transcriptome assembled de novo from sexual and asexual reads. (ZIP)

S2 Data Planarian genes encoding putative GPCRs in FASTA format. (FA)

S3 Data Planarian GPCR database; related to Fig 1. Classification results, annotations, and expression information corresponding to the GPCR genes identified in this work (see S2 Data). Column descriptions are as follows: **Clone ID:** Serial identifier used for database and classification purposes. **Gene name:** Names assigned to a number of genes by the authors. **#exons:** Number of exons detected based on mapping of GPCR genes to *Schmidtea mediterranea* genome version 2.0 [87]. The actual number of exons may be higher as some gene structures are incomplete. **Cluster:** Results of clustering analysis using CLANS 2.0. **Human:** Homologous human GPCR class. **BLAST description:** Top BLAST (protein vs. protein) hit against human proteins. **Lowest E-value:** E-value corresponding to the top BLAST hit against human proteins. **ORF confident:** Whether either end or both ends of the open reading frame are confidently discovered. **#TM domain:** Number of transmembrane domains detected by TMHMM 2.0. **Max. control RPKM:** Highest RPKM value resulting from mapping sexual or asexual RNA-seq reads to the GPCR sequence list. **Log2(FC(sex/asex)) [normalized]:** Log base 2 RPKM fold-change between sexual and asexual reads, normalized as described in methods. Values shown only if the associated *p*-value is smaller than 0.05. **Asex/Sex (Graveley):** RPKM fold-change between asexual and sexual RNA-seq reads from a previous study [88]. Values shown only if the associated *p*-value is smaller than 0.05. **Sexual irradiation (Graveley):** RPKM fold-change between irradiated and control sexual planarian RNA-seq reads [88]. Values shown only if the associated *p*-value is smaller than 0.05. **Asexual irradiation (Graveley):** RPKM fold-change between irradiated and control asexual planarian RNA-seq reads [88]. Values shown only if the associated *p*-value is smaller than 0.05. **X1 (SCs) (Pearson):** RPKM fold-change between X1 and differentiated sorted cell populations RNA-seq reads [89]. Values shown only if the associated *p*-value is smaller than 0.05. **X2 (Progeny) (Pearson):** RPKM fold-change between X2 and differentiated sorted cell populations RNA-seq reads [89]. Values shown only if the associated *p*-value is smaller than 0.05. **in situ pattern:** Expression patterns that were observed in at least two independent colorimetric ISH experiments. In all RPKM ratio values, “10,000” represents division by zero—i.e., no reads mapped to reference in the control sample. In **Log**_**2**_**(FC**_**sex/asex**_**) [normalized],** “13.29”, which is log_2_(10000), represents division by zero. In **in situ pattern,** the following abbreviations were used: BR, brain; INT, intestine; TE, testis; OV, ovary; OVD, oviduct; VIT, vitellaria; SPD, sperm duct; CO, copulatory apparatus; NE, no expression detected.
(XLSX)

S4 Data Quantification of FISH, RNAi, and receptor assay experiments. (XLSX)

S5 Data Primers used to clone the GPCRs analyzed. (XLSX)

S6 Data RNA-seq reads corresponding to the de novo assembly of GPCR genes. (ZIP)

S1 Fig Discovery and classification of planarian GPCRs; related to Fig 1. (A) Flowchart outlining identification of planarian GPCRs and subsequent follow-up analyses. See Materials and Methods for details. (B) Co-clustering of human, planarian, and other invertebrate GPCRs. Connections stronger than 1E-4 were considered for clustering. CLANS was run for 20,000 iterations. All planarian GPCRs are included and shown by solid blue circles. Human non-olfactory GPCRs (grey four-pointed stars) are used to map the main rhodopsin family and frizzled, glutamate, secretin, and adhesion receptors (all enclosed in dashed grey lines). Two planarian homologs of lung seven transmembrane receptors (LUSTR, GPR107 in humans) are indicated. Human and mouse olfactory receptors (pink four-pointed stars) cluster separately, and do not overlap with any planarian GPCRs. Similarly, no planarian GPCRs co-cluster with insect odorant receptors or chemoreceptors (six-pointed stars), or nematode chemoreceptors (heavy crosses). The only exception is the *srw* family of chemoreceptors that colocalizes with a group of planarian GPCRs. The *Rho-L* cluster neighbors amine receptors within the conserved rhodopsin family, suggesting that its members may retain affinity to small molecule ligands. Some members of *Srfb* have been previously identified as the PROF1 family of GPCRs [26]. (C) Neighbor-joining phylogenetic tree showing the hypothetical evolutionary relationship between planarian rhodopsin-like GPCRs. Conserved (D/E)R(Y/F) motifs are depicted in sequence logos. (D) Relative abundance of planarian GPCRs grouped according to their families or, in case of the rhodopsin family, separated by subfamilies. *Y*-axis shows RPKM values based on a mapping where only the GPCR database (and not a transcriptome) was used as the reference. For GPCRs that are differentially expressed between sexual and asexual strains, the higher values were used. Bars indicate the median and quartiles. GPCRs of the *Rho-L* subfamily are noticeably less abundant compared to the other groups. *Rho-R2* GPCRs are the most heterogeneous in terms of relative abundance. Frizzled and secretin GPCRs are on average the most abundant groups. (E) Bayesian inference topology of planarian NPY receptors with their closest counterparts throughout metazoans. Non-planarian GPCRs were selected only according to highest similarity in HMMER search (irrespective of the species of origin). Three types of planarian NPY receptors are identified: Type 1 including NPYR-1 to 6 and their arthropod and nematode homologs. *C*. *elegans* NPR-11 and *Drosophila* NPFR-1 are in this group. Type 2 includes planarian NPYR-8 to 10, in addition to many arthropod homologs. Type 3 includes planarian NPYR-11 to 16 and appears to be lophotrochozoan-specific. The snail NPY receptor GRL105 [40] is a member of this group. Vertebrate NPY receptors form a fourth monophyletic group that appears to be outside of the invertebrate clade (although with a lower 0.62 posterior probability). Posterior probabilities are 1.00 at every node, except those with a value shown. Common names or sequence identification numbers (GI) are shown for proteins on the tree. Tree is rooted with human and planarian amine receptors.
(TIF)

S2 Fig Planarian GPCRs are enriched in an assortment of tissues and organ systems; related to Fig 1. Representative colorimetric ISH experiments show GPCRs of different classes enriched in the nervous system, reproductive structures, and the intestine. (A) *gcr102* (unclustered) is expressed in a subset of cells in the ventral brain region (left) and putative sensory organs around the edge of the head on the dorsal side (right). (B) *gcr158* (*Rho-R2*) is expressed in cells associated with the cephalic ganglia. (C) *gcr121* (adhesion) is expressed in a handful of anterolateral cells. (D) *gcr106* (metabotropic glutamate receptor) is expressed both in the brain (left) and in the secretory glands around the copulatory apparatus (right). (E) *gcr084* (related to human transmembrane protein 181) is highly enriched in and around the penis papilla. (F) *gcr160* (*Rho-L1*) is expressed in a variety of epithelial tissues, including pharynx, seminal vesicles (left), around the head (middle), and the vitellaria (right). (G) *gcr153* (unclustered) in highly enriched in the intestine. (H–P) Expression patterns of representative NPY receptor genes. *npyr-1*, *3*, and *7* are expressed in subsets of cells in the brain. *npyr-2*, *5*, *6*, *8*, *9*, and *15* are enriched in the testes. *npyr-4* and *10* did not produce a specific ISH pattern. *npyr-11* to *14* and *16* were not tested or did not show specific expression. See S3 Data for a summary of expression patterns. Scale bars are 1 mm where whole animals are shown. Scale bars are 200 μm for insets.
(TIF)

S3 Fig Characterization of the *npyr-1* knockdown phenotype; related to Fig 2. (A) Double-FISH detects *nanos* (orange) and *gH4* (blue) expression in ovaries of control and *npy-8(RNAi)* worms. While control worms develop a complete ovary with mature oocytes (arrowheads), *npy-8(RNAi)* worms only display *nanos*^*+*^*/gH4*^*+*^ GSCs and *gH4*^*+*^ oogonia. Scale bars are 100 μm. (B) FISH labeling of *nanos* in *dmd-1(RNAi)* and *npyr-1(RNAi)* planarians. New *nanos*^*+*^ GSCs (orange) and *dmd-1*^*+*^ somatic testis cells (green in insets) are specified in regenerating *npyr-1(RNAi)* head fragments. *dmd-1(RNAi)* head fragments were used as controls. Although some cells expressing low levels of *dmd-1* can be detected in *dmd-1(RNAi)* regenerating worms, they were not able to re-specify *nanos*^*+*^ GSCs. Scale bars are 500 μm and 20 μm (insets). (C) qPCR experiments showing *npy-8* and *npyr-1* mRNA levels after four feedings of *npy-8* or *npyr-1* dsRNA in homeostatic mature sexuals. RNAi knockdown of *npy-8* or *npyr-1* only reduces the expression of the targeted gene. Neither knockdown significantly affects *nanos* expression. (D) qPCR experiments showing *npy-8* and *npyr-1* expression levels in sexual and asexual planarians. While *npy-8* is enriched ~50-fold in sexuals compared to asexuals, *npyr-1* is expressed at comparable levels across asexuals and hatchling and mature sexuals. Error bars in C and D are SEM for four individual worms in each treatment.
(TIF)

S4 Fig Validation of sexually enriched GPCRs by colorimetric ISH; related to Fig 4. Colorimetric ISH of representative sexually enriched GPCRs. *gcr108* (unclustered) is expressed in the inner layer of the testes, suggesting that *gcr108* expression is enriched in spermatids. Expression of 16 other GPCRs (*gcr124-141*; members of *Rho-L*, *Rho-C*, or *Srf/w*, or unclustered; *gcr140* was ruled out as a GPCR) are shown in the outer layer of the testes where spermatogonial cells are located. *gcr143* (unclustered) is expressed in the brain (top) as well as the vitellaria (bottom). *gcr144* (secretin) is expressed in the oviducts and copulatory apparatus. *gcr157* (*Srfb*) is enriched in the ovaries.
(TIF)

S5 Fig Low levels of *ophis* expression in asexual planarians; related to Fig 6. (A) FISH labeling *nanos* (orange), *dmd-1* (green), and *ophis* (magenta) in whole-mount asexual planarians. Clusters of *nanos*^*+*^ cells are present adjacent to *dmd-1*^*+*^ somatic cells on the dorsal side. *ophis* mRNA is not detectable in somatic cells. Imaging settings used were identical to other *ophis* FISH experiments. DAPI labels nuclei (grey). Scale bars are 100 μm. (B) qPCR analysis of *ophis* expression in asexual and hatchling and mature sexual planarians. Expression is comparable between asexuals and hatchling sexuals, but about 4-fold enriched in mature sexuals. Expression levels were averaged between four individual animals in each treatment and normalized to the expression level of *ophis* in asexual worms. Error bars represent SEM among biological replicates.
(TIF)

## Abbreviations

CNS: central nervous system
FISH: fluorescent in situ hybridization
FSH: follicle-stimulating hormone
GnRH: gonadotropin-releasing hormone
GPCR: G protein-coupled receptor
GSC: germline stem cell
ISH: in situ hybridization
LH: luteinizing hormone
PGC: primordial germ cell
