# Supplementary figures and images for: GPCRs Direct Germline Development and Somatic Gonad Function in Planarians
Source: PLoS Biol. 2016 May 10;14(5):e1002457. doi: 10.1371/journal.pbio.1002457 (PMC4862687; doi:10.1371/journal.pbio.1002457)

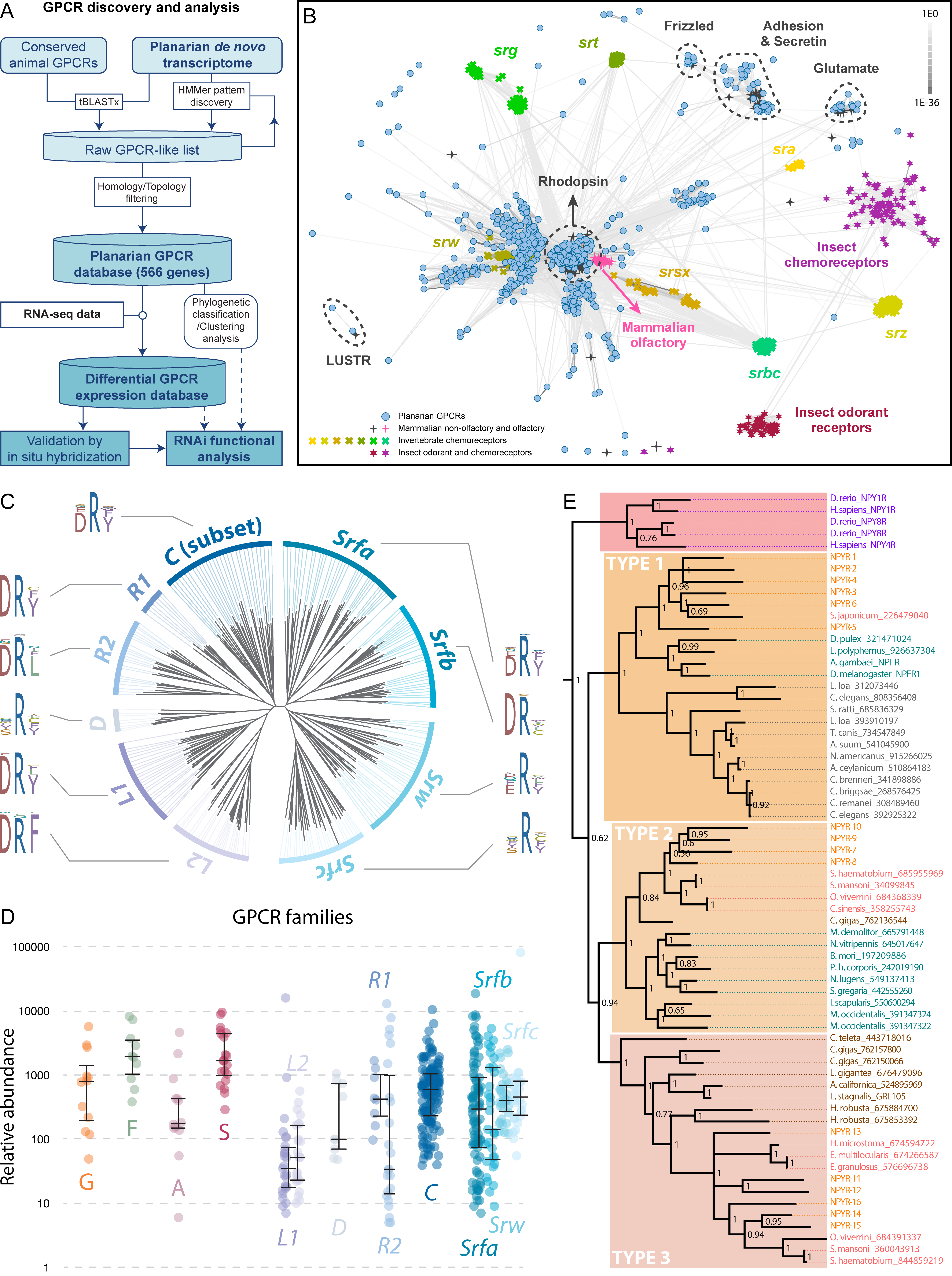

Supplement: S1 Fig — (A) Flowchart outlining identification of planarian GPCRs and subsequent follow-up analyses. See Materials and Methods for details. (B) Co-clustering of human, planarian, and other invertebrate GPCRs. Connections stronger than 1E-4 were considered for clustering. CLANS was run for 20,000 iterations. All planarian GPCRs are included and shown by solid blue circles. Human non-olfactory GPCRs (grey four-pointed stars) are used to map the main rhodopsin family and frizzled, glutamate, secretin, and adhesion receptors (all enclosed in dashed grey lines). Two planarian homologs of lung seven transmembrane receptors (LUSTR, GPR107 in humans) are indicated. Human and mouse olfactory receptors (pink four-pointed stars) cluster separately, and do not overlap with any planarian GPCRs. Similarly, no planarian GPCRs co-cluster with insect odorant receptors or chemoreceptors (six-pointed stars), or nematode chemoreceptors (heavy crosses). The only exception is the srw family of chemoreceptors that colocalizes with a group of planarian GPCRs. The Rho-L cluster neighbors amine receptors within the conserved rhodopsin family, suggesting that its members may retain affinity to small molecule ligands. Some members of Srfb have been previously identified as the PROF1 family of GPCRs [26]. (C) Neighbor-joining phylogenetic tree showing the hypothetical evolutionary relationship between planarian rhodopsin-like GPCRs. Conserved (D/E)R(Y/F) motifs are depicted in sequence logos. (D) Relative abundance of planarian GPCRs grouped according to their families or, in case of the rhodopsin family, separated by subfamilies. Y-axis shows RPKM values based on a mapping where only the GPCR database (and not a transcriptome) was used as the reference. For GPCRs that are differentially expressed between sexual and asexual strains, the higher values were used. Bars indicate the median and quartiles. GPCRs of the Rho-L subfamily are noticeably less abundant compared to the other groups. Rho-R2 GPCRs ar [file pbio.1002457.s007.tif]

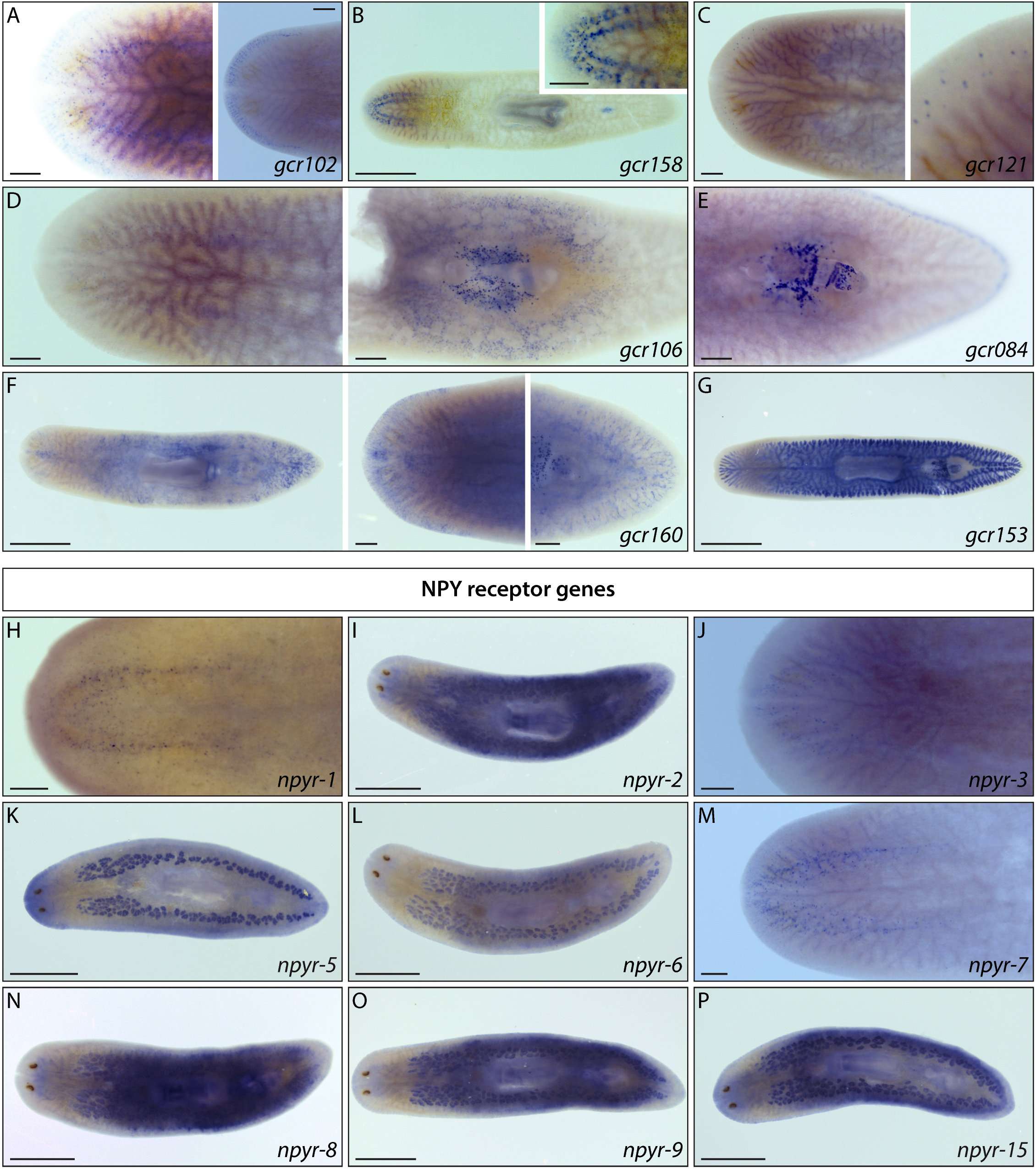

Supplement: S2 Fig — Representative colorimetric ISH experiments show GPCRs of different classes enriched in the nervous system, reproductive structures, and the intestine. (A) gcr102 (unclustered) is expressed in a subset of cells in the ventral brain region (left) and putative sensory organs around the edge of the head on the dorsal side (right). (B) gcr158 (Rho-R2) is expressed in cells associated with the cephalic ganglia. (C) gcr121 (adhesion) is expressed in a handful of anterolateral cells. (D) gcr106 (metabotropic glutamate receptor) is expressed both in the brain (left) and in the secretory glands around the copulatory apparatus (right). (E) gcr084 (related to human transmembrane protein 181) is highly enriched in and around the penis papilla. (F) gcr160 (Rho-L1) is expressed in a variety of epithelial tissues, including pharynx, seminal vesicles (left), around the head (middle), and the vitellaria (right). (G) gcr153 (unclustered) in highly enriched in the intestine. (H–P) Expression patterns of representative NPY receptor genes. npyr-1, 3, and 7 are expressed in subsets of cells in the brain. npyr-2, 5, 6, 8, 9, and 15 are enriched in the testes. npyr-4 and 10 did not produce a specific ISH pattern. npyr-11 to 14 and 16 were not tested or did not show specific expression. See S3 Data for a summary of expression patterns. Scale bars are 1 mm where whole animals are shown. Scale bars are 200 μm for insets. (TIF) [file pbio.1002457.s008.tif]

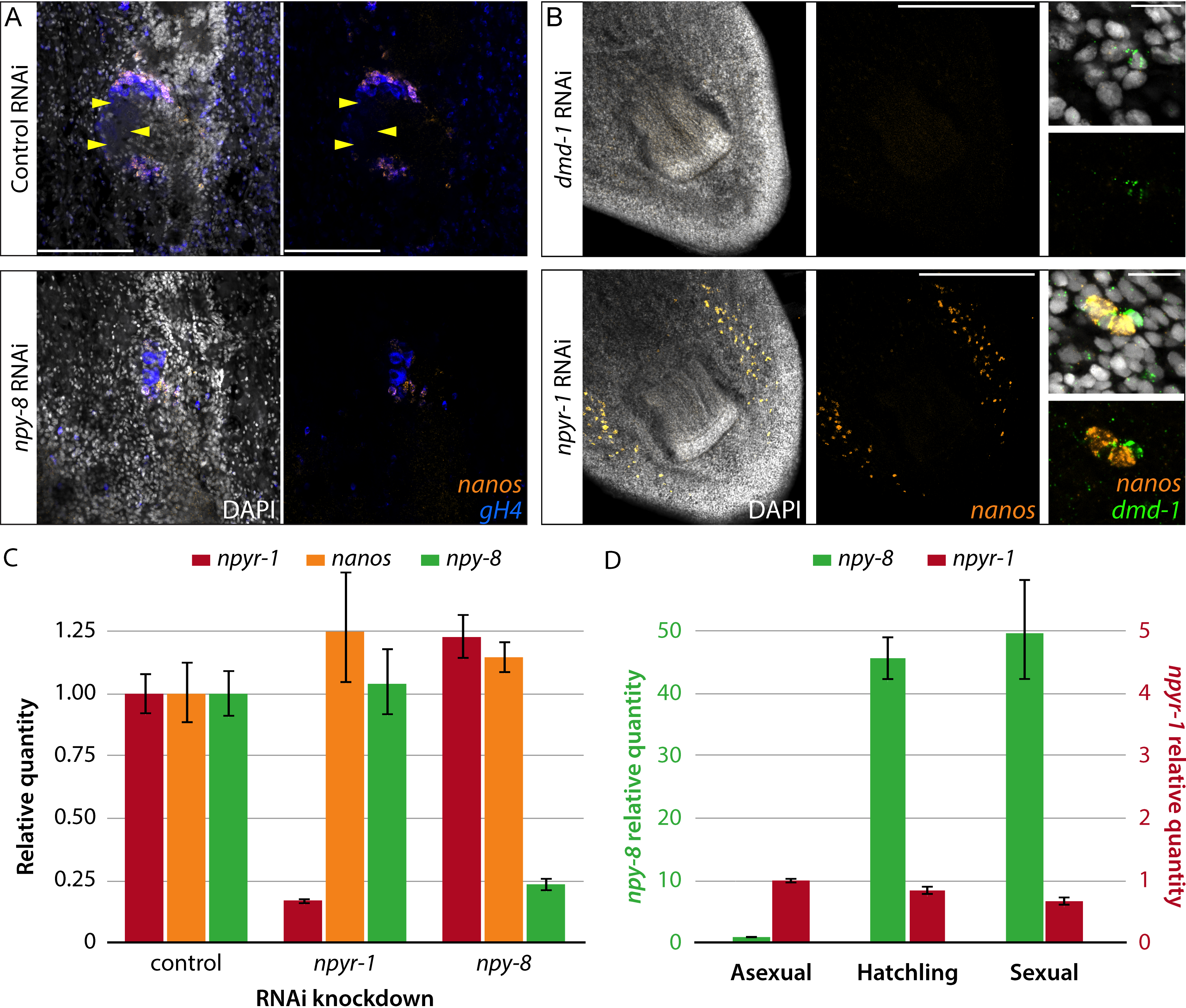

Supplement: S3 Fig — (A) Double-FISH detects nanos (orange) and gH4 (blue) expression in ovaries of control and npy-8(RNAi) worms. While control worms develop a complete ovary with mature oocytes (arrowheads), npy-8(RNAi) worms only display nanos+/gH4+ GSCs and gH4+ oogonia. Scale bars are 100 μm. (B) FISH labeling of nanos in dmd-1(RNAi) and npyr-1(RNAi) planarians. New nanos+ GSCs (orange) and dmd-1+ somatic testis cells (green in insets) are specified in regenerating npyr-1(RNAi) head fragments. dmd-1(RNAi) head fragments were used as controls. Although some cells expressing low levels of dmd-1 can be detected in dmd-1(RNAi) regenerating worms, they were not able to re-specify nanos+ GSCs. Scale bars are 500 μm and 20 μm (insets). (C) qPCR experiments showing npy-8 and npyr-1 mRNA levels after four feedings of npy-8 or npyr-1 dsRNA in homeostatic mature sexuals. RNAi knockdown of npy-8 or npyr-1 only reduces the expression of the targeted gene. Neither knockdown significantly affects nanos expression. (D) qPCR experiments showing npy-8 and npyr-1 expression levels in sexual and asexual planarians. While npy-8 is enriched ~50-fold in sexuals compared to asexuals, npyr-1 is expressed at comparable levels across asexuals and hatchling and mature sexuals. Error bars in C and D are SEM for four individual worms in each treatment. (TIF) [file pbio.1002457.s009.tif]

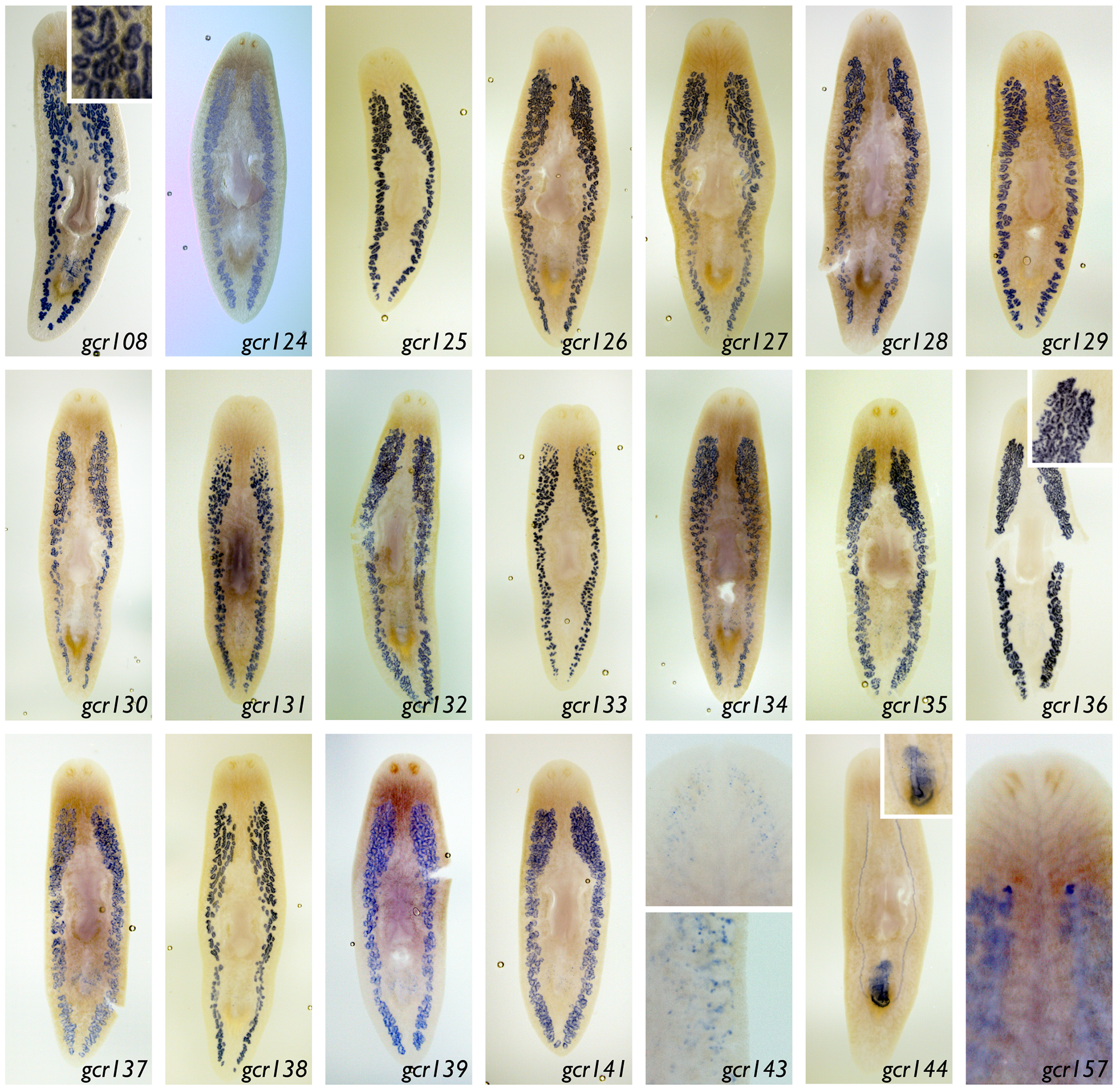

Supplement: S4 Fig — Colorimetric ISH of representative sexually enriched GPCRs. gcr108 (unclustered) is expressed in the inner layer of the testes, suggesting that gcr108 expression is enriched in spermatids. Expression of 16 other GPCRs (gcr124-141; members of Rho-L, Rho-C, or Srf/w, or unclustered; gcr140 was ruled out as a GPCR) are shown in the outer layer of the testes where spermatogonial cells are located. gcr143 (unclustered) is expressed in the brain (top) as well as the vitellaria (bottom). gcr144 (secretin) is expressed in the oviducts and copulatory apparatus. gcr157 (Srfb) is enriched in the ovaries. (TIF) [file pbio.1002457.s010.tif]

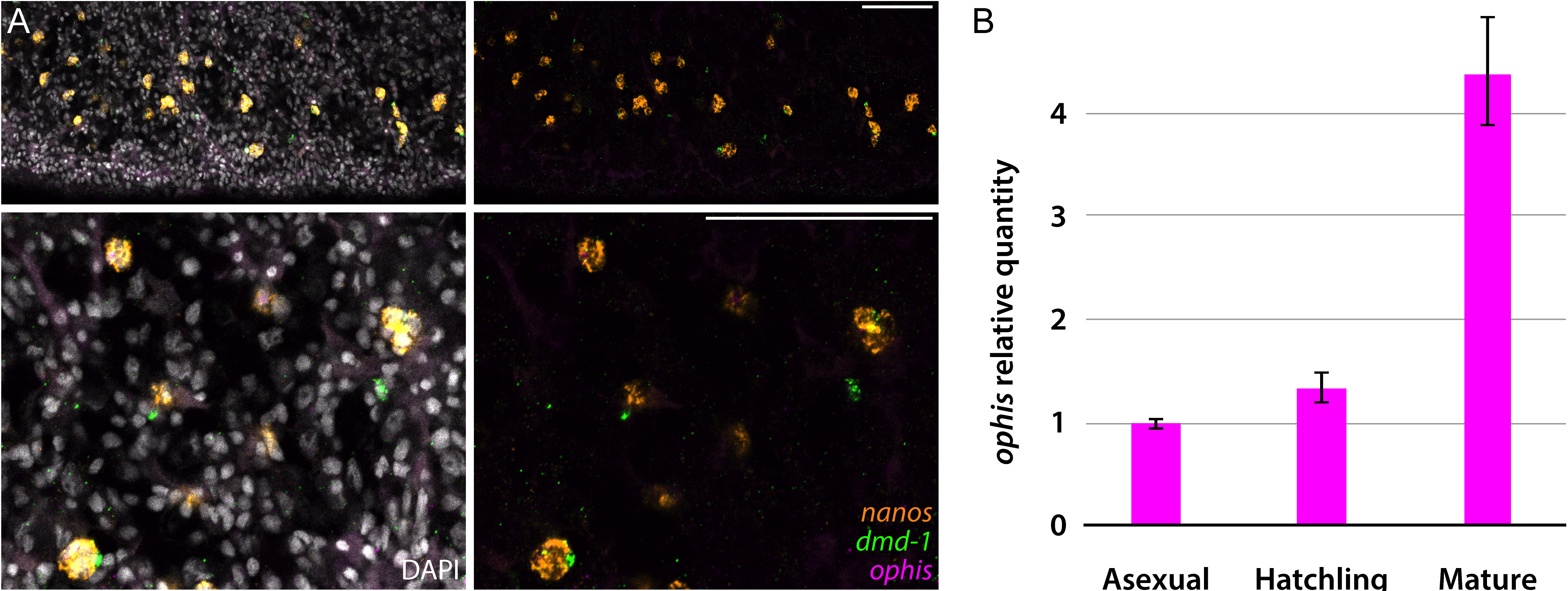

Supplement: S5 Fig — (A) FISH labeling nanos (orange), dmd-1 (green), and ophis (magenta) in whole-mount asexual planarians. Clusters of nanos+ cells are present adjacent to dmd-1+ somatic cells on the dorsal side. ophis mRNA is not detectable in somatic cells. Imaging settings used were identical to other ophis FISH experiments. DAPI labels nuclei (grey). Scale bars are 100 μm. (B) qPCR analysis of ophis expression in asexual and hatchling and mature sexual planarians. Expression is comparable between asexuals and hatchling sexuals, but about 4-fold enriched in mature sexuals. Expression levels were averaged between four individual animals in each treatment and normalized to the expression level of ophis in asexual worms. Error bars represent SEM among biological replicates. (TIF) [file pbio.1002457.s011.tif]
